# Nonswelling Lubricative Nanocolloidal Hydrogel Resistant to Biodegradation

**DOI:** 10.1007/s40820-025-01830-0

**Published:** 2025-07-11

**Authors:** Tiantian Ding, Chunxia Ren, Liyuan Meng, Guoyong Han, Yao Xue, Wenlong Song, Daowei Li, Hongchen Sun, Bai Yang, Yunfeng Li

**Affiliations:** 1 State Key Laboratory of Supramolecular Structure and Materials, College of Chemistry, Jilin University, 2699 Qianjin Street, Changchun, 130012 People’s Republic of China; 2 Jilin Provincial Key Laboratory of Oral Biomedical Engineering, Hospital of Stomatology, Jilin University, Changchun, 130021 People’s Republic of China; 3 Joint Laboratory of Opto-Functional Theranostics in Medicine and Chemistry, The First Hospital of Jilin University, Changchun, 130021 People’s Republic of China

**Keywords:** Nanocolloidal hydrogel, Nonswelling, Degradation resistance, Low friction

## Abstract

Hydrogels derived from biopolymers have numerous applications in bioengineering, drug delivery, wound healing, and wearable devices. Yet, their strong swelling and uncontrollable degradation stimulate the development of hydrogels that overcome these limitations. Here, we report nanocolloidal hydrogels formed from nanoparticles of methacryloyl-modified biopolymers that exhibit resistance to swelling and enzymatic degradation both in vitro and in vivo, along with exhibiting a broad-range of mechanical and lubrication properties, wear resistance and biocompatibility. The nonswelling behavior of nanocolloidal hydrogels takes origin in the resistance to swelling of their hydrophobic regions which are resulted from the nanophase of hydrophobic methacryloyl groups in the interior of the constituent nanoparticles. The developed approach to the preparation of nanocolloidal hydrogel with greatly enhanced properties will have applications in long-term drug delivery and cell culture, soft tissue augmentation, and implantable bioelectronics.

**Supplementary Information:**

The online version contains supplementary material available at 10.1007/s40820-025-01830-0.

## Highlights

- Novel nanocolloidal hydrogels form nanoparticles of methacryloyl-modified biopolymers.
- The nanocolloidal hydrogels exhibit tunable mechanical properties, nonswelling behavior, excellent biodegradation resistance, and good biocompatibility.
- The nanocolloidal hydrogels show outstanding lubrication with a friction coefficient as low as ~ 0.0018.

**Supplementary Information:**

The online version contains supplementary material available at 10.1007/s40820-025-01830-0.

## Graphical abstract

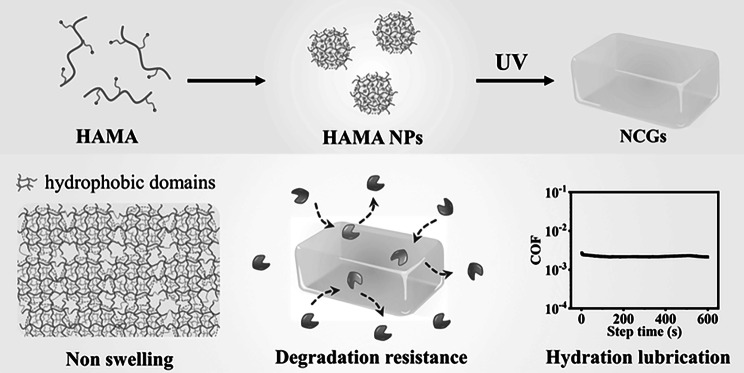

## Introduction

Hydrogels are soft viscoelastic materials usually composed of a three-dimensional (3D) network that is inflated by water [1–4]. Because of the high fraction of water and the ability to control the composition, permeability, mechanical properties, and biocompatibility of hydrogels, they find a broad range of applications in drug and cell delivery, wound dressings, wearable devices, and tissue regeneration [1–5]. Generally, hydrogels have a greater osmotic pressure than the surrounding aqueous environment, which leads to their swelling [6]. This effect can be advantageous in controlled drug delivery [7], programmable shape-morphing [8], atmospheric water harvesting [9], and expansion microscopy [10], however, it greatly changes hydrogel’s mechanical properties and permeability [1, 11]. Such changes limit hydrogel applications in long-term cell culture [3], drug delivery [7], and implantable bioelectronics [12, 13]. For example, swelling may cause compression and damage of tissue when a hydrogel is applied in cranium or spinal cord [14, 15]. Swelling of the hydrogel used for the sealing of the annulotomy caused postoperative cauda equina syndrome [14]. Furthermore, hydrogel degradation further amplifies its swelling, causing side effects in clinical hydrogel applications [16]. For instance, swelling caused by the degradation of the commercialized hydrogel MIRAgel used for the treatment of retinal detachment resulted in strong compression of the eyeball, potentially causing blindness of a patient [16–18].

The limitations caused by hydrogel swelling have been addressed by several strategies. For example, strong hydrogel crosslinking reduced the extent of its swelling [19–21], however, this approach generally yielded brittle hydrogels [22, 23]. The use of polymers with hydrophobic fragments [24], or the incorporation of thermoresponsive components with a lower critical solution temperature (LCST) in the hydrogel network was utilized to regulate its hydrophilic-lipophilic balance and hence, reduce hydrogel swelling [11, 25, 26]. Yet, this approach is difficult to control because the hydrophilic-lipophilic balance could be easily shifted when hydrogel composition or ambient conditions change [27]. Recently, additional strategies have been proposed to mitigate the swelling of hydrogels through interesting molecular and structural design [28], including the use of architectures of brush polymers with extended chain conformations prior to gelation [26], preparation of double-network hydrogels with a stiff armor [29], and the addition of hydrophobic fillers in the hydrogels [30, 31]. These approaches have shown promise in mitigating swelling, however, they showed limitations in achieving a long-term stability, biocompatibility, and mechanical robustness simultaneously. These constraints stimulate the design and synthesis of nonswelling hydrogels with controlled mechanical strength, adjustable component concentrations, and temperature-independent nonswelling behavior for their translational applications in bioengineering, bioelectronics, soft tissue augmentation, and drug delivery.

Recently, nanocolloidal hydrogels (NCGs) have emerged as a new class of soft matter, in which nanoparticles (NPs) act as physically or the chemically crosslinked building blocks of the network swollen with water [32, 33]. NCGs can exhibit structural hierarchy, nonlinear mechanics, interesting optical properties [32], thixotropy [34, 35], and enhanced and modulated transport properties [36–39]. Because of these properties, NCGs have already found applications as optical coatings [40, 41], biosensors [42], absorbents [43], and materials for soft robotics [44] and bioengineering [32]. Despite these advances, nonswelling biocompatible NCGs with long-term resistance to degradation have not been reported.

Herein, we report a nonswelling NCG derived from methacryloyl hyaluronate (HAMA) NPs formed by the nanoprecipitation method. In the interior of these NPs, the methacryloyl groups associated in the nanoscopic hydrophobic domains, which were subsequently photocrosslinked to form poly(methyl methacrylate) oligomer. The NCG prepared by the photocrosslinked HAMA NPs exhibited a temperature-independent nonswelling behavior in a broad range of NP concentration and degrees of HAMA modification with methacryloyl groups. The nonswelling of the NCG was attributed to the resistance to swelling of the HAMA NP building blocks. The NCGs showed biocompatibility and long-term resistance to biodegradation after 6-month long subcutaneous implantation in mice. In addition, the NCGs exhibited lubrication against a variety of materials with coefficient of friction (COF) as low as ~ 0.0018. Because of these advanced properties, the developed NCG has many promising biomedical applications.

## Experimental Section

### Materials

Sodium hyaluronate (HA, *Mw*, 150,000–250,000 g mol^−1^), gelatin (type B), and FITC-dextran (*Mw*, 70,000 g mol^−1^) were purchased from Shanghai Yuanye Bio-Technology Co., Ltd. Methacrylic anhydride (MA), adipic dihydrazide (ADH), and guanidine hydrochloride were purchased from Aladdin. Additionally, phosphate buffer saline (PBS 10.0 mM, pH = 7.4) and glutaraldehyde (25 wt%) were purchased from Macklin. 1-(3-dimethylaminopropyl)-3-ethyl carbodiimide hydrochloride (EDC) was purchased from TCI. 2-Hydroxy-4’-(2-hydroxyethoxy)-2-methylpropiophenone (Irgacure 2959), sigmacote, sodium hydroxide (NaOH), hyaluronidase (Type I-S, lyophilized powder, 400–1,000 units mg^−1^), and sodium dodecyl sulfate (SDS) were obtained from Sigma-Aldrich. Moreover, Arg-Gly-Asp (RGD) peptides modified with methacryloyl group were purchased from Wuhan Holder Co., Ltd. Ultra-pure water was prepared by RSJ Water Purification System (China).

### Preparation of HAMA NPs

To synthesize methacryloyl hyaluronate (HAMA), 2–5 mL of MA was added to 100 mL solution of 10.0 mg mL^−1^ HA. The pH of the solution was adjusted to 8.0–9.0 using 5.0 M NaOH and the reaction was allowed to take place for 24 h at ~ 0-4 °C [45]. After that, the mixed solution was purified by the reverse precipitation method in cold anhydrous ethanol. The precipitates were dissolved in deionized water and the solution was dialyzed against deionized water through a dialysis membrane (MWCO, 12-14 kDa) for at least 72 h, with water being changed three times every day. The final product was obtained by lyophilization and stored at −20 °C. ^1^H nuclear magnetic resonance spectroscopy was used to quantify the degree of substitution of HA. The lyophilized HAMA was dissolved in deuterium water (D_2_O, 5.0 mg mL^−1^) and the solution was analyzed using a 500-MHz NMR spectrometer (Bruker AVANCEIII500, USA).

In the procedure of the preparation of the HAMA NPs, 0.4 g HAMA was dissolved in 80 mL of deionized water. Following this step, 136 mL of acetone was added into the HAMA solution and the mixed solution was stirred for 30 min at room temperature. Subsequently, 160.0 mg EDC and 80.0 mg ADH were introduced into the solution. After stirring for 30 min, 131 mL acetone was added, and the solution was stirred for 3–5 h. After that, the mixed solution was dialyzed against deionized water using a dialysis membrane (MWCO, 12-14 kDa) for at least 72 h, with the water being exchanged three times a day. The dispersion of HAMA NPs was concentrated using a rotary evaporator. Finally, the HAMA NPs were dispersed in 10.0 mM PBS (pH = 7.4) with a certain concentration. The syntheses of HAMA NPs with different sizes were identical to the method described above, except that the feeding ratios between the added adipic dihydrazide and the carboxyl groups of HAMA were adjusted to 50% and 10%.

### Characterization of HAMA NPs

The morphology of HAMA NPs was examined by transmission electron microscopy (TEM, JEM-2100, Japan) operating at an accelerating voltage of 200 kV. A droplet (2 μL) of 1.0 mg mL^−1^ suspension of HAMA NPs was placed on the copper grid with carbon support film. The diameter of HAMA NPs was characterized by measuring their dimensions using the software ImageJ. Dynamic light scattering experiments (DLS, Zetasizer Pro, Malvern Instruments Ltd.) were used to measure the hydrodynamic diameter and the electrokinetic potential (ζ-potential) of HAMA NPs with* C*_NPs_ = 1.0 mg mL^−1^ in deionized water. The degree of cross-linking of HAMA NPs by adipic dihydrazide was determined by elemental analysis (Elementar Vario micro cube). The viscosity of the HAMA NPs in PBS was assessed by using a rheometer (Discovery HR-10, TA Instruments, USA) equipped with a parallel plate and a Peltier plate with crosshatched surface (*d* = 20.0 mm). The viscosity of 30.0 mg mL^−1^ HAMA and 30.0 mg mL^−1^ HAMA NPs was tested at 37 °C, using the rheometer equipped with the temperature controller.

### Preparation of Methacryloyl Gelatin NPs

Gelatin powder (type B, 5.0 g) was dissolved in 100 mL of deionized water at 50 °C. Then, 100 mL of acetone was added to the solution, and the resulting suspension was left to stand at room temperature for 1 h. After removing the supernatant, the gelatin precipitate was dissolved in 50 mL of deionized water again at 50 °C. The purified gelatin was obtained using freeze-drying. To prepare gelatin NPs, 3.8 g of lyophilized gelatin was dissolved in 75 mL of deionized water at 50 °C with the pH being further adjusted to 2.5. Subsequently, 225 mL of acetone was slowly added to the solution at a rate of 12 mL min^−1^. After cooling to room temperature, 555 μL of the glutaraldehyde solution (25 wt%) was added to the gelatin dispersion to crosslink gelatin NPs. After 24 h, 300 mL of the guanidine hydrochloride solution (100 mM) was added to the dispersion. The resulting dispersion was stirred for 1 h to block the unreacted aldehyde groups. Gelatin NPs were purified through the 4-time centrifugation (10,000 r min^−1^, 1 h). To functionalize the gelatin NPs with methacryloyl groups, the gelatin NPs were dispersed in a 0.1 M sodium bicarbonate buffer and stirred evenly at 50 °C. The pH of the solution was then adjusted to 9.0 by 5.0 M NaOH. Subsequently, methacrylic anhydride was added to the mixture at a ratio of 1.2 mL per gram of gelatin NPs. The reaction was stirred for 1 h at 50 °C. After the reaction, the mixed solution was dialyzed against deionized water through a dialysis membrane (MWCO, 12-14 kDa) for at least 72 h and the water was changed three times every day. After the dialysis, the pH was adjusted to 7.4. Finally, gelatin NPs were obtained by freeze drying and stored at -20 °C. ^1^H NMR spectroscopy was used to quantify the degree of substitution of methacryloyl group in methacryloyl gelatin NPs by using 0.1 wt% of sodium dodecyl sulfonate (SDS) as a chemical shift reference. The methacryloyl gelatin NPs contained 0.18 mmol of methacryloyl groups per gram of NPs.

### Preparation of NCGs

The precursor in PBS (10.0 mM, pH = 7.4) for the NCGs included HAMA NPs with various concentrations and 0.05% (w/v) Irgacure 2959. Subsequently, 600 μL of precursor was added into a circle polytetrafluoroethylene (PTFE) mold (20.0 mm × 20.0 mm). The precursor in the PTFE mold was then exposed to the UV light irradiation (Uvitron, 365 nm, 10 mW cm^−2^) for 2 min to obtain the NCGs. For the synthesis of NCGs of gelatin at a concentration of 150.0 mg mL^−1^, the methacryloyl gelatin NPs were dissolved in PBS (10.0 mM, pH = 7.4) containing 0.5% (w/v) Irgacure 2959 to prepare the precursor. The synthesis steps for NCGs were identical to the above process. The precursor was polymerized under the UV light irradiation (Uvitron, 365 nm, 10 mW cm^−2^) for 5 min to prepare NCGs of gelatin. To prepare binary NCGs, 0.4 mL of 30.0 mg mL^−1^ HAMA NPs, 0.1 mL of 100.0 mg mL^−1^ methacryloyl gelatin NPs, and 0.1 mL of 0.3% (w/v) Irgacure 2959 solution were evenly mixed. Subsequently, the mixed dispersion was polymerized under UV light irradiation (365 nm, 10 mW cm^−2^) for 2 min.

### SAXS Characterization of NPs and NCGs

The SAXS profiles of HAMA NPs, methacryloyl gelatin NPs, and corresponding NCGs were conducted on an Xeuss3.0 instrument equipped with Cu as radiation sources. The light tube power was 30 W and an X-ray with a wavelength of 1.54 Å was used as the radiation source with the beam area of 0.9 mm × 0.9 mm. The distance between samples to detector was 400 mm. Each SAXS pattern was collected within 60 min. The SAXS data were analyzed by software Xenocs XSACT 2.7.

### Characterization of Transverse Relaxation Times (*T*_2_)

The *T*_2_ was determined by Carr–Purcell–Meiboom–Gill (CPMG) NMR measurements. The sweep width and O1 were set as 10 and 3 ppm, respectively. The recovery time was set as 10, 13, 20, 28, 35, 45, 65, 100, 300, 600, 1000, and 1500 ms. The protons of methacryloyl groups on HAMA NPs were chosen for analysis by integrating the peak area in the software of Origin. The area of the NMR peak of methacryloyl motifs from HAMA NPs at each recovery time was fitted with a double-exponential decay to obtain the* T*_2_.

### Transmission, Permeability, and Structure Characterization of NCGs

To measure the transmission of NCGs, we prepared the NCGs with a thickness of 1.0 mm between two glass slides. The transmission of NCGs was measured using the transmission mode of the Shimadzu 3100 UV–Vis spectrophotometer instrument with two glass slides as a reference.

To characterize the diffusion and transport characteristics of NCGs, the FITC-dextran (*Mw*, 70,000 g mol^**−1**^) was encapsulated in NCGs at a concentration of 1.0 mg mL^−1^. NCGs containing FITC-dextran were then incubated in 3 mL of PBS (10.0 mM, pH = 7.4) at 37 °C on an orbital shaker. At selected time points, 50 μL of the PBS solution was removed and analyzed to obtain fluorescent intensity of the solution on the Elisa reader (Bio-Tek, Synergy LX, USA). The concentration of FITC-dextran was determined from the fluorescence intensity by a standard curve. To calculate the diffusion coefficients of FITC-dextran in NCGs to the PBS, a semi-infinite plate approximation was used.

1 $${\text{C}}_{{{\text{media}}}} {\text{ = C}}_{{{\text{matrix}}}} { }\frac{{{\text{A}}_{{{\text{matrix}}}} }}{{{\text{V}}_{{{\text{bath}}}} }}{2}\sqrt {{\text{t}}\frac{{{\text{D}}_{{{\text{matrix}}}} }}{{\uppi }}}$$

where C_matrix_ is the initial concentration of encapsulated FITC-dextran and A_matrix_ is the surface area of the NCGs through which flux occurs. D_matrix_ is the diffusion coefficient within NCG matrices which was calculated from the slope of linear curves of C_media_ versus the square-root of t [46, 47].

The structure of the NCGs was evaluated by cryogenic scanning electron microscopy (cryo-SEM, Zeiss SIGMA 300 field-emission, Oberkochen, Germany) equipped with a cryo-system (PP3010, Quorum Technologies Ltd., East Sussex, UK). The NCGs were cut into small pieces (1.0 cm × 2.0 mm × 1.0 mm). These pieces were frozen in liquid nitrogen and placed inside the sample holder. Subsequently, the NCGs were broken by a scraper, sublimated to remove ice, and sprayed with platinum in the cryo-SEM sample preparation station. Finally, the samples were transferred to the SEM chamber and imaged at an accelerating voltage of 3.0 kV [48].

### Volume Swelling of NCGs

We used NCG rods to measure their volume swelling. The NCG rods were prepared in capillaries with an inner diameter of ~ 0.9–1.1 mm. The inner surface of the glass capillary was hydrophobic treated with sigmacote to remove NCG rods from the capillary easily. The NCG rods (25.0 mg mL^−1^ HAMA NPs in 10.0 mM PBS) were prepared by UV light irradiation (10 mW cm^−2^, 365 nm) for 2 min. The NCG rods were carefully removed from the glass capillary and gently transferred to a petri dish with a diameter of 3.5 cm. The initial diameter of the NCG rods, D_0_, was measured using an optical microscope (Nikon, Eclipse Ts2-FL). Subsequently, 5 mL PBS (10.0 mM, pH = 7.4) was added to the petri dishes containing NCG rods to study the volume swelling of the NCGs. The diameter of the NCGs at selected time points (D_x_) was measured using an optical microscope. Based on an assumption that hydrogels deform isotropically, the volume swelling ratio of NCG rods was calculated according to the following equation [27]:

2 $${\text{Volume}}\;{\text{swelling}}\;{\text{ratio}} = \left( {\frac{{{\text{D}}_{{\text{X}}} }}{{{\text{D}}_{0} }}} \right)^{3} \times 100\%$$

To investigate the effect of PBS concentrations on NCG swelling, the NCG rods were incubated in PBS solutions of 20.0, 50.0, and 100.0 mM at 25 °C for 200 days. The diameter of the NCG rods was measured at selected time points. Furthermore, to study the influence of the temperature on NCG swelling, the NCG rods were incubated in 10.0 mM PBS for 200 d at 4, 25, and 37 °C, respectively. The diameter of the NCG rods was measured at selected time points. The volume swelling ratio was calculated using the Eq. (2). The method for measuring the volume swelling of NCGs of methacryloyl gelatin NPs and binary mixture of HAMA NPs and methacryloyl gelatin NPs was identical to the method mentioned above.

### Mass Swelling of NCGs

The NCG discs with a diameter of 10.0 mm and a thickness of 1.0 mm were prepared in a PTFE mold containing 25.0 mg mL^−1^ HAMA NPs in 10.0 mM PBS under the UV light illumination (10 mW cm^−2^, 365 nm) for 2 min. Subsequently, the NCG discs were carefully transferred from the PTFE mold to a petri dish of 3.5 cm in diameter. The initial mass of the NCG discs was denoted as M_0_. A 5.0 mL PBS (10.0 mM, pH = 7.4) was added to petri dishes containing NCG discs to study the mass swelling of the NCGs. The mass of the NCG discs was measured at 1, 2, 3, 4, 5, 6, and 7 days, respectively, after removing the PBS solution from the petri dish and gently wiping the surface of the NCGs with Kimwipes. The equilibrium mass of the NCGs was denoted as M_x_. The mass swelling ratio was calculated according to the following equation.

3 $$ {\text{Mass}}\;{\text{swelling}}\;{\text{ratio}} = \frac{{{\text{Mx}}}}{{{\text{M}}_{0} }} \times 100\% $$

To investigate the effect of PBS concentrations on the NCG swelling, the NCGs were incubated at 25 °C for 7 days in PBS solutions of 20.0, 50.0, and 100.0 mM, respectively. The mass of the NCG discs was measured daily. Furthermore, to study the influence of the temperature on the NCG swelling, the NCGs were incubated in 10.0 mM PBS solution for 7 days at 4, 25, and 37 °C, respectively. The mass of the NCGs was measured daily. The swelling ratio was calculated using the Eq. (3).

### Mechanical Properties and Lubricating Properties of NCGs

The mechanical properties of NCGs were assessed using a rheometer (Discovery HR-10, TA Instruments, USA) equipped with a parallel plate and a Peltier plate with crosshatched surface (d = 20.0 mm). The storage modulus and loss modulus of NCGs were determined by strain and frequency sweep experiments at 37 °C. The strain sweep experiments were performed with the oscillation strain ranging from 0.1% to 100.0% at a constant frequency of 0.1 Hz. Frequency sweep experiments were performed with a frequency ranging from 0.1 to 100.0 Hz at 1.0% strain.

To measure their COF, the NCGs of 2.0 mm in thickness and 22.0 mm in diameter were prepared. The COF of the NCGs was evaluated by a rheometer (Discovery HR-10, TA Instruments, 25 °C). At the shear rate of 0.1 s^−1^, the axial forces of 0.1, 0.3, 0.5, and 1.0 N, respectively, were applied to the NCGs to assess their friction properties. When the axial force was 0.3 N, the friction properties of the NCGs were examined at shear rates of 0.25, 0.5, and 1.0 s^−1^, respectively. The measurement duration was 300.0 s, during which torque (Г) and axial force (N) were collected. The COF value was calculated by the following equation [49]:

4 $${\text{COF}} = \frac{4\Gamma }{{3{\text{RN}}}}$$

After the friction test, the NCG was removed from the rheometer and the morphology of the NCGs was examined using a Nikon D7500 camera. The microscopic changes in the surface morphology of the hydrogel were observed using the microscope (Nikon, Eclipse Ts2-F). To evaluate NCG friction properties, the rheometer test of the NCGs were performed for 6 h at initial axial force of 1.0 N and shear rate of 0.1 s^−1^. The 6 h measurement was repeated four times. After each test, the morphology of the hydrogels was inspected using a Nikon D7500 camera, while the microscopic changes in the surface morphology were observed under the microscope (Nikon, Eclipse Ts2-F). The COF of the NCGs against to different materials was also measured using rheometry (Discovery HR-10, TA Instruments, USA). Disc of different materials with a diameter of 20.0 mm and a thickness of 2.0 mm were prepared and was fixed on the upper plate of the rheometer with the double-sided adhesive. The NCG was placed on the lower plate of the rheometer. The friction test was performed at a shear rate of 0.1 s^−1^ and an axial force of 0.3 N. Unless stated otherwise, the solvent trap was utilized to minimize water evaporation in all the measurements.

### Cytocompatibility of NCGs

Mouse L929 fibroblast cells and rabbit hyaline chondrocyte cells were cultured in Dulbecco’s modified Eagle’s medium (DMEM; Gibco, Carlsbad, CA, USA) containing 10% (v/v) fetal bovine serum (FBS; HyClone Laboratories, Inc., Logan, UT USA) and 1% (v/v) penicillin–streptomycin solution in an incubator with 5% CO_2_ at 37 °C. To assess the cytocompatibility NCGs, mouse L929 fibroblast cells and rabbit hyaline chondrocyte cells were cultured in 3D NCGs, respectively. In brief, 30,000 L929s or hyaline chondrocyte cells were encapsulated in NCGs by copolymerization with 0.1% Arg-Gly-Asp (RGD) peptides modified with methacryloyl group, 25.0 mg mL^−1^ HAMA NPs, and 0.05% Irgacure 2959 in a 24-well plate. After 3 days, the medium was removed, and the NCG was rinsed twice with PBS (10.0 mM, pH = 7.4). Subsequently, 250 μL of Calcein/PI was added into each well. The L929s or hyaline chondrocytes were stained for 20 min at 37 °C. Fluorescent images of cells were captured using an optical microscope (BX53, Olympus, Tokyo, Japan).

### Hemolysis Experiment of NCGs

Red blood cells (RBCs) were collected from the fresh mouse blood and suspended in PBS (10.0 mM, pH = 7.4) at the density of 1 × 10^7^ cells mL^−1^. Subsequently, the RBC suspensions were incubated with NCGs, saline (as a negative control), and 1 wt % Triton X-100 (used as a positive control) for 3 h at 37 °C. After that, the suspensions were centrifuged at 2000 r min^−1^ for 10 min. The absorbance of supernatant was measured at 545 nm using UV–visible spectroscopy. The hemolysis ratio of material (%) was calculated as:

5 $$\frac{{{\text{[A]t}} - {\text{[A]n}}}}{{{\text{[A]p}} - {\text{[A]n}}}} \times {1}00\%$$

where [A]_t_, [A]_p_, and [A]_n_ were the absorbance values of the sample, positive, and negative groups, respectively.

### In Vitro and In Vivo Degradation Behavior of NCGs

In vitro NCG degradation was examined by measuring the change in dimensions during hydrogel incubation with the hyaluronidase. Hydrogel rods were prepared in a glass capillary with inner diameter of 0.9–1.1 mm, evacuated and gently transferred to a 3.5 cm diameter petri dish. A 5 mL hyaluronidase solution in PBS (10.0 mM, pH = 7.4) with a concentration of ~ 200–500 units mL^−1^ was added to the NCG rods in the petri dish. Optical microscopy images of NCGs were acquired at selected time points at room temperature. The diameter of the NCG rods was measured by the software ImageJ. The hyaluronidase solution in PBS was replaced every 48 h.

To investigate the in vivo degradation of NCGs, three NCG discs with a diameter of 4.7 mm and a thickness of 1.0 mm were prepared by using 25.0 mg mL^−1^ HAMA-28 NPs dispersion. The discs were implanted at various locations on the back of Balb/C mice. The samples were collected at 1, 2, 4, 8, 16, and 24 weeks post-implantation. During each sampling, the mice were sacrificed, the mouse skin and the NCGs were collected, the photographs of the NCG disks were taken using the camera (Nikon D7500). The diameter of the NCG disks at the implantation position was measured. At each time points, there were three laboratory mice and three control mice.

All animal experiments were conducted in accordance with the Guidelines for the Care and Use of Laboratory Animals of Jilin University and approved by the Animal Ethics Committee of Jilin University (approval number: 20230521).

### H&E Staining

The collected organs and tissues (heart, liver, spleen, lung, kidney, and skin surrounding the implant sites) were fixed in 4% paraformaldehyde at 4 °C for 24 h. The fixed tissues were dehydrated in a graded series of ethanol (80%, 90%, and 100% ethanol in water). Subsequently, the samples were embedded in paraffin and cut into 3 μm-thick sections. The sections were stained with H&E and examined by two independent senior pathologists using an optical microscope (BX53, Olympus, Tokyo, Japan).

### Inflammatory Extent Grade

Inflammatory cell levels were scored using a semiquantitative scale. Six representative areas of fibrous capsules in each section were determined to assess quantitatively the fibrous capsule quality. The locations were scored based on the number and location of fibroblasts and inflammatory cells present in each specimen [50, 51].

### Immunostaining of Tissue Sections and Quantitative Image Analysis

Tissue sections were first deparaffinized and rehydrated, followed by immunohistochemical staining performed according to the manufacturer's instructions. The antibodies for TNF-α, iNOS, and IL-1β were diluted at a ratio of 1:200. The percentage of the area occupied by TNF-α, iNOS, and IL-1β positive regions relative to the total tissue area was quantified using ImageJ software.

### Statistical Analysis

All data were expressed as mean ± SD. Student's t-tests and two-way ANOVA analysis were performed to evaluate the statistical significance among the groups. *P* < 0.05 was regarded as statistical significance.

## Results and Discussion

### Preparation and Structural Characterization of NCGs

**Fig. 1:**
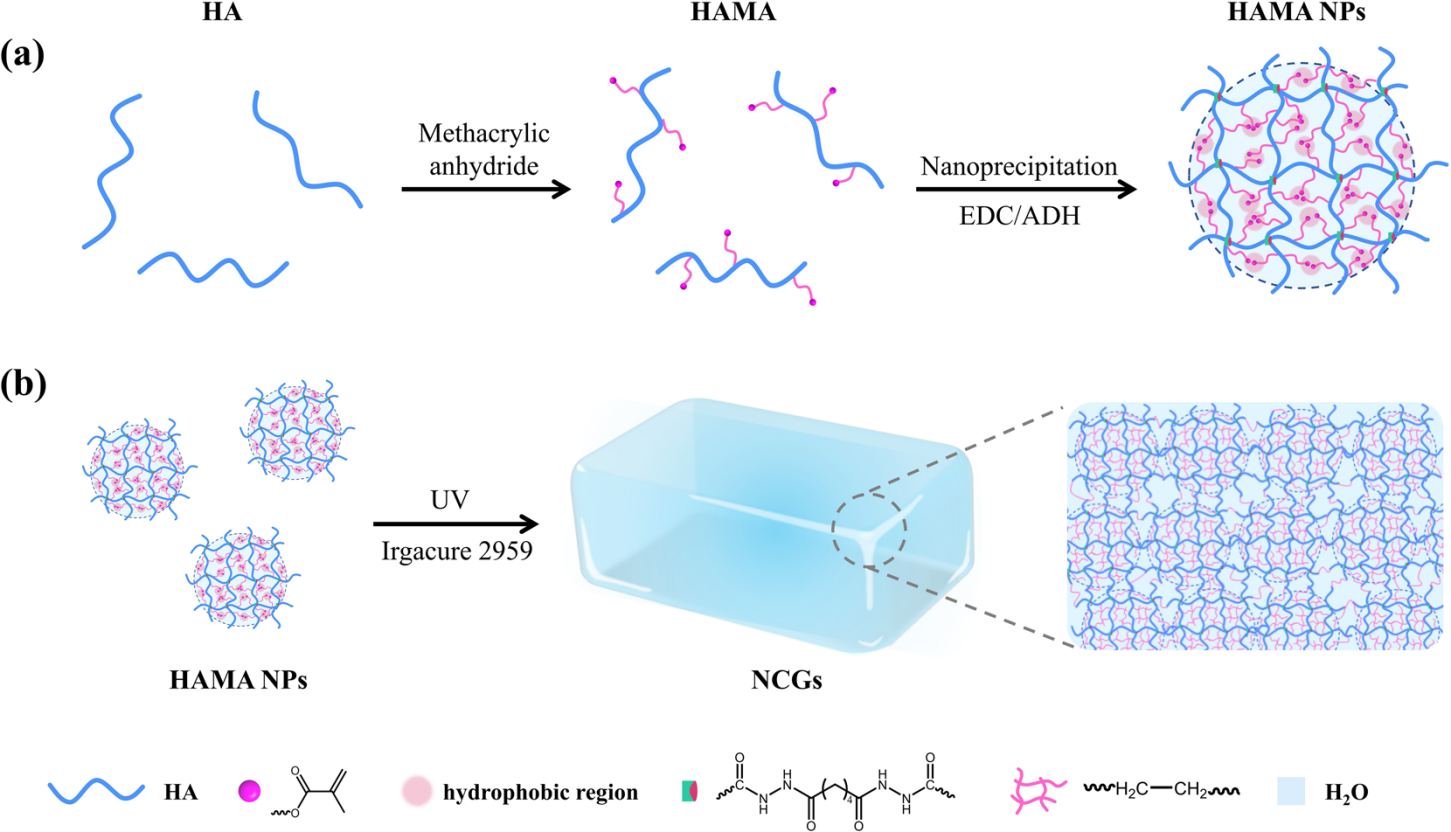
Preparation of NCGs. **a** Preparation of HAMA NPs using a nanoprecipitation method. **b** Preparation of NCG by NP photocrosslinking using a photoinitiator and UV light. ADH represents adipic dihydrazide

Figure 1 illustrates the design and preparation of the NCG. First, the reaction of sodium hyaluronate with methacrylic anhydride for the formation of HAMA was confirmed by ^1^H nuclear magnetic spectrum (NMR) experiments (Fig. S1a). The degree of substitution of methacryloyl groups, DS, was controlled from 15% to 35% by changing the feeding mass ratios of methacrylic anhydride-to-sodium hyaluronate (Fig. S1b). The NPs were prepared via two-step nanoprecipitation method [52] by mixing an aqueous HAMA solution and acetone under stirring and subsequently, chemically crosslinking the NPs by the reaction between the carboxyl groups on HAMA and adipic dihydrazide in the presence of 1-(3-dimethylaminopropyl)-3-ethyl carbodiimide hydrochloride (EDC) [53] (Fig. 1). Here, we refer to HAMA NPs with a different DS of methacryloyl groups as HAMA-R NPs, where R is equal to 100 × DS. The degree of NP crosslinking was determined by elemental analysis (Table S1). No significant change in the degree of crosslinking of HAMA NPs was observed when the DS increased from 15% to 35% (Table S2).

**Fig. 2:**
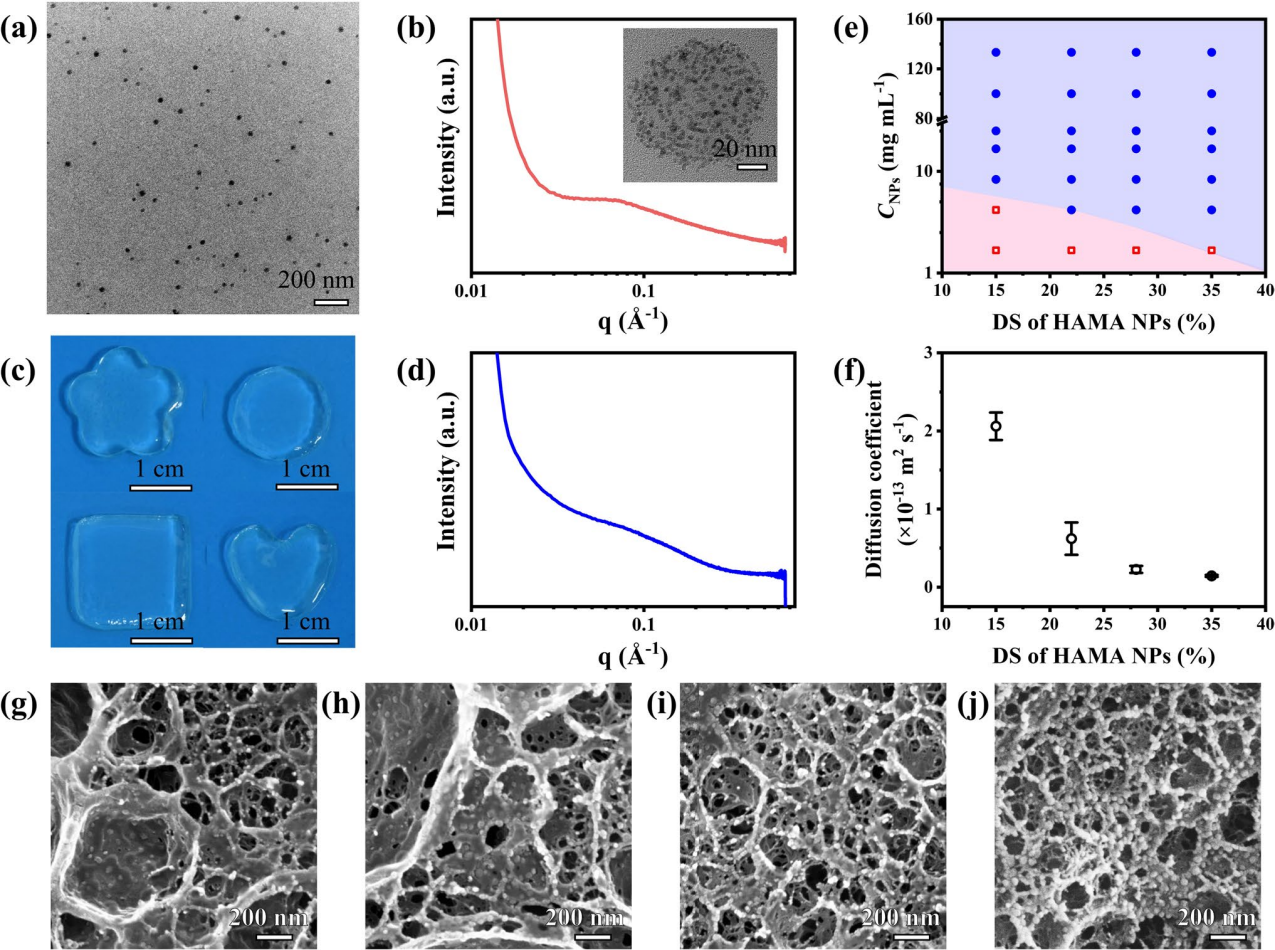
NCGs prepared from HAMA NPs. **a** TEM image of HAMA-28 NPs. **b** SAXS profile of HAMA-28 NPs in water. Inset shows the TEM image of HAMA-28 NPs stained by ammonium tetrachloropalladate. **c** Photographs of NCGs with different shapes. **d** SAXS profile of NCGs made from 25.0 mg mL^−1^ HAMA-28 NPs. **e** State diagram of the NCGs by using HAMA NPs with different DS and *C*_NPs_. **f** Variation in diffusion coefficient of 70,000 g mol^−1^ FITC-dextran in the NCGs made from 25.0 mg mL^−1^ HAMA NPs with different DS. Error bars represent standard deviations based on three independent measurements. **g-j** Cryo-SEM images of NCGs made from 25.0 mg mL^−1^ HAMA NPs with the DS of **g** 15%, **h** 22%, **i** 28%, and **j** 35%

Figure 2a shows a representative TEM image of spherical shape HAMA-28 NPs with diameter of 26.0 ± 4.3 nm. Based on TEM image analysis, the NPs maintained their diameter at ~ 25.0 nm with the DS increasing from 15% to 35% (Fig. S2a–f). The average hydrodynamic diameter and electrokinetic potential (ζ-potential) of HAMA NPs dispersed in deionized water were approximately 200.0 nm and − 25.0 mV with DS increasing from 15% to 35% (Fig. S3a, b). The dimensions of HAMA NPs were controlled by varying the ratio between the added adipic dihydrazide and the carboxyl groups of HAMA (Fig. S4a, b). When this ratio increased from 10 to 100%, the hydrodynamic diameter of HAMA NPs decreased from 911 ± 80 to 171 ± 67 nm, respectively, while ζ-potential reduced from − 41.5 ± 4.7 to − 22.9 ± 4.5 mV (Fig. S4c, d). The corresponding diameter of HAMA NPs determined by TEM image analysis decreased from ~ 292.6 ± 51.5 to 26.0 ± 4.3 nm. In comparison with dry NPs for TEM imaging, the NPs strongly swelled in water, which resulted in their larger size in the DLS measurement.

Interestingly, the association of hydrophobic methacryloyl groups caused nanophase separation in the HAMA NP interior. The existence of hydrophobic domains was characterized by small angle X-ray scattering (SAXS) (Figs. 2b and S5). The peak of ~ 0.07 Å^−1^ in the SAXS profiles confirmed the existence of spherical domains in the HAMA NP interior. After fitting the SAXS data [54], the radius of gyration (R_g_) of these regions was found to be ~ 0.7 nm when DS increased from 15 to 35% (Note S1 and Table S3). Accordingly, these segregated regions had radius of ~ 1.0 nm. The segregation of methacryloyl groups in the NP interior was further visualized as black inclusions by staining them with ammonium tetrachloropalladate and TEM imaging the HAMA NPs (Inset of Figs. 2b and S5). Moreover, the measurements of transverse relaxation times (T_2_) in ^1^H NMR experiments revealed that the NPs exhibited two T_2_ values (Fig. S6), with a smaller T_2_ ≈ 20–30 ms corresponding to the interior of the HAMA NPs. The larger T_2_ ≈ 120–160 ms ascribed to the polymer chains at the NP exterior, suggesting the existence of dangling chains on the surface of HAMA NPs (Note S2). The HAMA NPs did not aggregate in water at the concentration, *C*_NPs,_ of up to 160.0 mg mL^−1^. In contrast, linear HAMA with the same molecular weight was only dissolved to 50.0 mg mL^−1^ in an aqueous solution [45]. The viscosity of HAMA NPs dispersion in phosphate buffered saline (PBS) was significantly lower than that of the solution of linear HAMA with the same concentration (Fig. S7).

Next, to form an NCG, an aqueous dispersion of HAMA NPs containing a photoinitiator was exposed to UV irradiation for 2 min to trigger crosslinking of methacryloyl groups between the NPs and in the NPs. The NCGs exhibited transmittance over 95% in the range from 300 to 800 nm (Figs. 2c and S8). Based on the results of SAXS, the ~ 2 nm-size hydrophobic domains in the HAMA NP interior were preserved in the NCGs (Figs. 2d, S9, and Table S3). Figure 2e shows the diagram characterizing NCG formation in PBS, plotted by varying *C*_NPs_ and DS of HAMA NPs. Generally, the use of HAMA NP dispersions with a high concentrations or NP with high DS favored the NCG formation. A stable NCG was prepared when the *C*_NPs_ increased from 4.2 to 133.3 mg mL^−1^.

Transport properties of the NCG were characterized by encapsulating 70,000 g mol^−1^ FITC-dextran in the hydrogel and subsequently measuring its diffusion into the surrounding PBS. The diffusion coefficient of FITC-dextran decreased from 2.06 × 10^–13^ ± 0.18 × 10^–13^ to 1.44 × 10^–14^ ± 0.13 × 10^–14^ m^2^ s^−1^ as the DS of HAMA NPs increased from 15% to 35% (Fig. 2f). The cryogenic scanning electron microscopy (cryo-SEM) was further used to examine the NCG structure. Figure 2g–j shows the cryo-SEM images of the hydrogel made from HAMA NPs with different DS. The HAMA NPs were clearly observed in the porous network (Fig. S10). Based on the analysis of cryo-SEM images, the size of pores in the NCGs decreased gradually from 108 ± 47 to 35 ± 11 nm as the DS of HAMA NPs increased from 15% to 35%. Moreover, the crosslinking density of the NCGs increased from 53.0 ± 1.6 to 192.1 ± 6.3 mol m^−3^ with the DS increasing from 15% to 35% (Note S3 and Table S4). The increase in the crosslinking density of the NCGs led to the decrease of diffusion coefficient and pore size. The fraction of water in the NCGs was evaluated by measuring the loss of water after the NCG drying (Table S4).

### Nonswelling Properties of NCGs

**Fig. 3:**
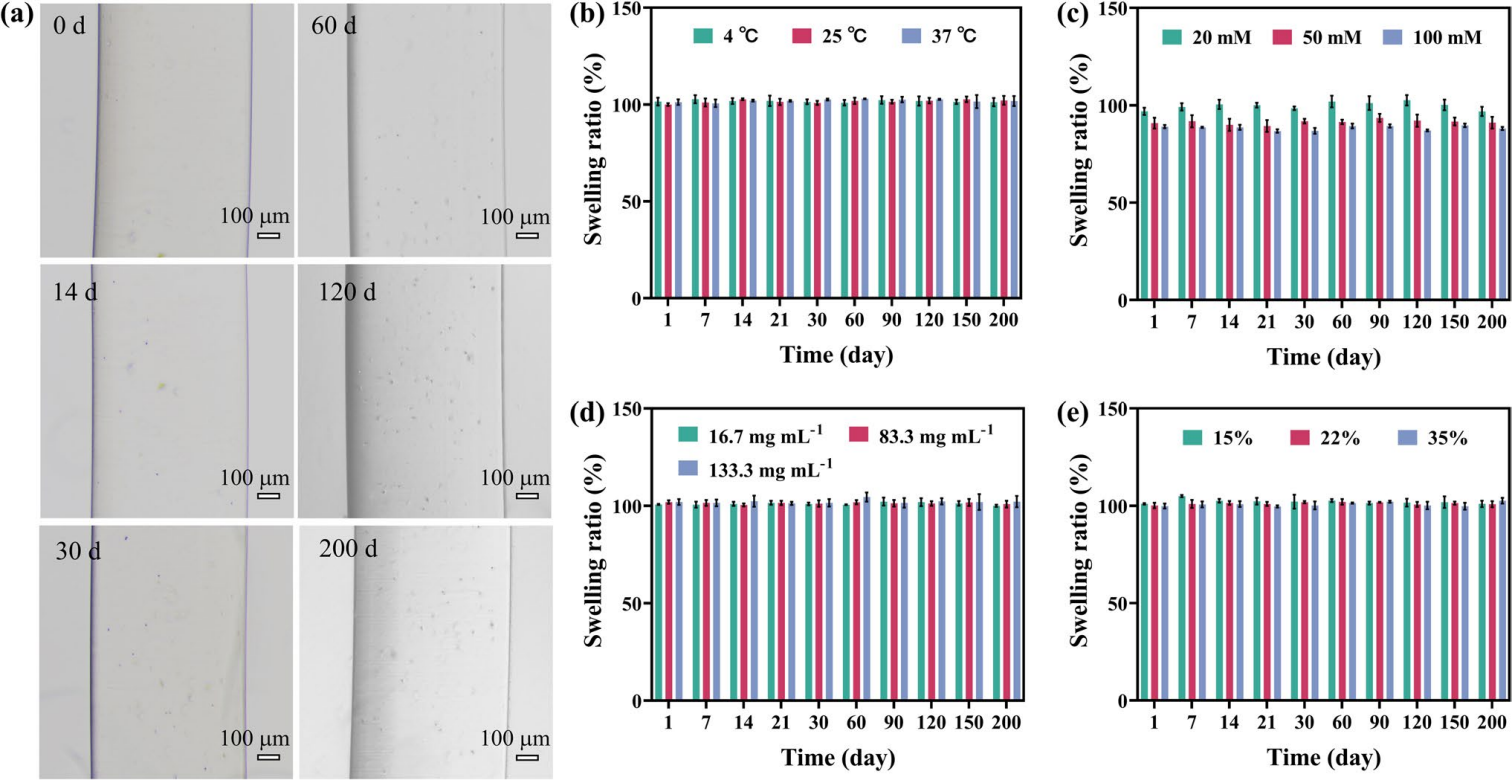
Nonswelling properties of NCGs. **a** Representative optical microscopy images of NCGs formed from HAMA-28 NPs after incubation in PBS (10.0 mM, pH = 7.4) for 200 days at 25 °C. **b** Swelling ratio of NCGs made from HAMA-28 NPs in 10.0 mM PBS at 4, 25, and 37 °C. **c** Swelling ratio of NCGs made from HAMA-28 NPs in 20.0, 50.0, and 100.0 mM PBS solution at 25 °C. The *C*_NPs_ in a-c was 25.0 mg mL^−1^. **d** Swelling ratio of NCGs made from 16.7, 83.3, and 133.3 mg mL^−1^ HAMA-28 NPs in 10.0 mM PBS solution at 25 °C. **e** Swelling ratio of NCGs made from 25.0 mg mL^−1^ HAMA NPs with DS of 15%, 22%, and 35% at 25 °C. Error bars represent standard deviations based on three independent measurements

The swelling behavior of the NCG was examined by measuring its swelling ratio, (D_t_/D_0_)^3^, upon immersion in PBS (10.0 mM, pH = 7.4, 25 °C) for up to 200 days, where D_t_ and D_0_ is the diameter of the NCG rod at the selected time point and its initial diameter, respectively. Figure 3a shows that the NCG rods formed by HAMA-28 NPs exhibited no noticeable change in dimensions after 200 days in PBS. Moreover, because no thermoresponsive segments were used in the hydrogel, the ratio (D_t_/D_0_)^3^ ≈ 1.0 of the rods was observed at 4, 25, and 37 °C (Fig. 3b). Furthermore, the NCGs resisted deswelling in PBS with concentration from 10.0 to 100.0 mM (Figs. 3c and S11a-d). The resistance to deswelling of NCGs in the PBS with higher ionic strength might be attributed to the unique structure of densely packed NPs and dual crosslinking networks in hydrogels. These structural features might restrict the mobility and collapse of polymer chains in hydrogels, leading to a high elastic pressure and thus the resistance to deswelling in hypertonic solution [27]. The NCGs exhibited nonswelling behavior independent of the *C*_NPs_, that is, their volume swelling ratio was ~ 1.0 as the *C*_NPs_ increased from 16.7 to 133.3 mg mL^−1^ (Fig. 3d). Moreover, the NCGs remained nonswelling regardless of the DS of HAMA NPs (Figs. 3e and S12a–c). Notably, after being immersed in PBS for 200 days at 25 °C, the NCGs maintained their shape and size, which indicated their stability against hydrolysis. Note that the NCGs prepared by HAMA NPs with different dimensions were also nonswelling (Fig. S13).

The nonswelling behavior of NCGs was further confirmed by measuring their masses at selected time points (Fig. S14). The mass swelling ratio, M_x_/M_0_, was used to evaluate the swelling behavior of NCGs, where M_x_ was the mass of the NCG at the selected time point and M_0_ was the initial mass of the NCG. After immersion in PBS (10.0 mM, pH = 7.4) for 7 days, nonswelling was observed for the NCGs derived from HAMA-28 NPs (Fig. S14a), independently of the temperatures and the DS of the NPs (Fig. S14b, c). Similarly, as the PBS concentration increased from 10.0 to 100.0 mM, the mass swelling ratio of NCGs decreased only from ~ 1.0 to ~ 0.9 (Fig. S14d). The superior nonswelling characteristics of NCGs can be further demonstrated through comparing their swelling ratio with those of nonswelling or antiswelling hydrogels reported in the literature (Table S5).

The nonswelling behavior of the NCG in a broad range of conditions was attributed to the resistance to swelling of the HAMA NPs carrying photocrosslinked hydrophobic regions in their interior. To this end, the swelling of individual HAMA NPs in a very dilute dispersion was measured before and after UV cross-linking. After photocrosslinking of the methacryloyl groups completely (Fig. S15a), nanoscopic hydrophobic domains in the HAMA NP interior were preserved, which was determined by SAXS (Fig. S16). These nanophase domains exhibited the resistance to swelling of the individual HAMA NPs, leading to nonswelling of HAMA NPs upon photocrosslinking (Fig. S15b). The exposure of the dispersion of HAMA NPs in PBS with added photoinitiator to UV irradiation led to the formation of the NCGs due to the photoinitiated reaction between the methacryloyl groups on the NP surface and concurrently, photocrosslinking of the methacryloyl groups with the formation of the hydrophobic nanodomains of poly(methyl methacrylate) in the NP interior, which was confirmed by SAXS (Figs. 2d and S9).

To further confirm this mechanism of nonswelling, a hydrogel made from linear HAMA as well as an NCG formed from HA NPs modified by aldehyde-group and gelatin NPs were prepared as control systems. The hydrogels prepared from HAMA exhibited swelling with a swelling ratio of ~ 1.6 (Fig. S17a). This result suggested that the formation of the hydrophobic crosslinks between the HAMA molecules did not completely suppress hydrogel swelling. Colloidal hydrogel prepared from HA NPs modified by aldehyde-group and gelatin NPs through imine crosslinking also swelled with a swelling ratio of ~ 1.2 (Fig. S17b). No hydrophobic nanophase existed in both HA NPs modified by aldehyde-group and gelatin NPs. This result suggested that the hydrophobic nanophase in the NCGs played a crucial role in the resistance to swelling.

The versatility of the engineering approach to nonswelling NCGs was evaluated by forming gels from gelatin NPs. Methacryloyl gelatin NPs (Fig. S18a, b) were prepared by the nanoprecipitation method and subsequently used to prepare NCGs as illustrated in Fig. 1 (Fig. S18c). A weak peak of ~ 0.04 Å^−1^ in the SAXS profiles confirmed nanophase separation in the interior of methacryloyl gelatin NP with the domain radius of ~ 0.6 nm (Fig. S18d). After the NCG formation, the hydrophobic poly(methyl methacrylate) nanodomains with a radius of ~ 1.3 nm were formed in the constituent NPs, which was confirmed by SAXS (Fig. S18d). As a result, the NCGs formed from methacryloyl gelatin NPs showed a nonswelling behavior (Fig. S19a, b). The nonswelling NCGs were also prepared from NP mixtures, that is, from HAMA NPs and methacryloyl gelatin NPs (Fig. S19c, d).

### Hydrogel Mechanical Properties

**Fig. 4:**
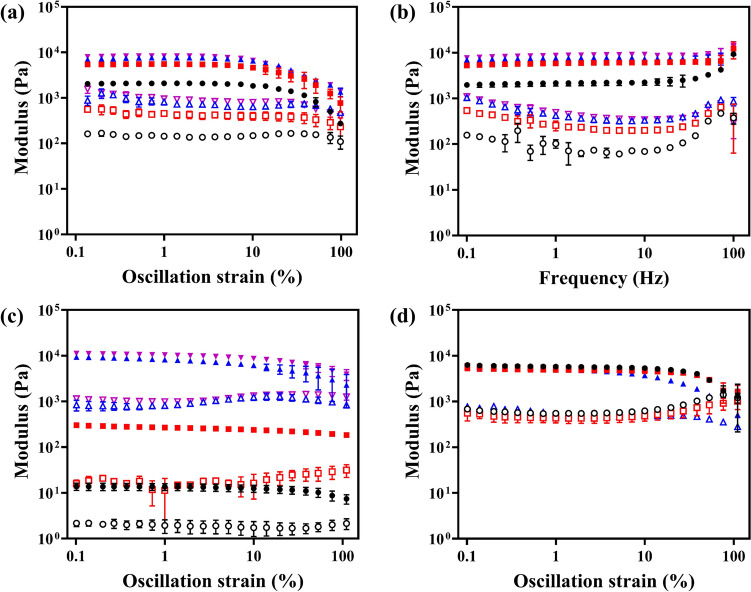
Mechanical properties of NCGs. **a**, **b** Storage modulus, G′, and loss modulus, G′′ of NCGs made from 25.0 mg mL^−1^ HAMA NPs with DS of 15% (black), 22% (red), 28% (blue), and 35% (pink) in **a** strain and **b** frequency sweep experiments performed at 37 °C. **c** G′ and G′′ of NCGs made from HAMA-28 NPs with *C*_NPs_ of 4.2 (black), 8.3 (red), 83.3 (blue) and 133.3 mg mL^−1^ (pink) conducted at 37 °C. **d** The modulus changes of NCGs with 25.0 mg mL^−1^ HAMA-28 NPs immersed in PBS for 7 (red), 15 (blue), and 30 d (pink). Solid and open symbols represent storage and loss modulus, respectively. Error bars represent standard deviations based on three independent measurements

Strain and frequency sweep rheological experiments were performed for the NCGs at 37 °C to determine their linear viscoelastic behavior (Fig. 4a, b). At < 10.0% strain, the NCGs showed a linear viscoelastic response (Fig. 4a), with the storage modulus, G′ being greater than the loss modulus, G′′. At > 10.0% strain, the NCGs showed a nonlinear viscoelastic behavior, with both G′ and G′′ decreasing as the strain increased (Fig. 4a). The decrease in moduli was caused by the fracture of hydrogel. In the frequency range of 0.1–100.0 Hz, the NCGs exhibited a linear viscoelastic response with the storage modulus, G′ > G′′ (Fig. 4b). The mechanical properties of the NCGs were controlled by the DS of HAMA NPs and *C*_NPs_. For example, at 5% strain, the value of G′ of the NCGs increased from 2014 ± 59 to 7687 ± 406 Pa as the DS increased from 15% to 35%, respectively (Fig. 4a). The value of G′of the NCGs increased from 14 ± 3 to 11,128 ± 218 Pa with *C*_NPs_ increasing from 4.2 to 133 mg mL^−1^, respectively (Fig. 4c). The shear moduli of the NCGs almost matched the mechanical properties of the most of the soft tissues [55, 56]. To further evaluate NCG stability, the rheological measurements were conducted for the NCG prepared from HAMA-28 NPs after immersing it in PBS for 7, 15, and 30 days. Figure 4d shows that the rheological properties of the NCGs did not change. Moreover, the NCGs prepared from HAMA NPs with DS of 15%, 22%, and 35% all exhibited invariant mechanical properties after 30-day immersion in PBS (Fig. S20a-c).

### Hydrogel Lubrication Properties

**Fig. 5:**
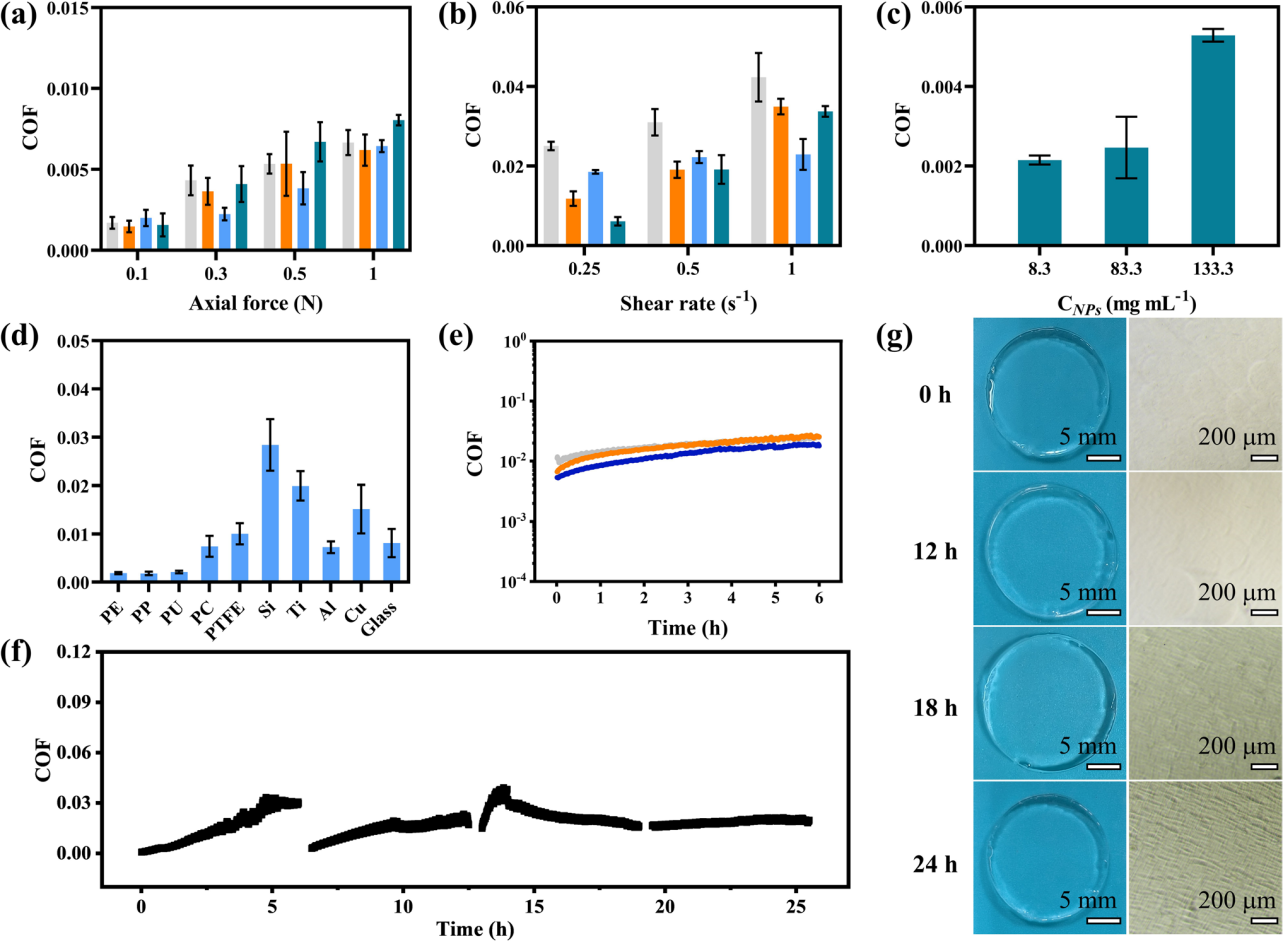
Lubricating properties of NCGs. **a** Variation in COF of NCGs measured at 25 °C under axial forces in the range of 0.1 to 1.0 N at the constant shear rate of 0.1 s^−1^. **b** Variation in COF of NCGs measured at 25 °C under shear rate in the range of 0.25 to 1.0 s^−1^ at the constant axial force of 0.3 N. The NCGs in **a** and **b** were prepared from 25.0 mg mL^−1^ HAMA NPs with the DS of 15% (gray), 22% (orange), 28% (blue), and 35% (green). **c** Variation in COF of NCGs as a function of concentrations of the HAMA-28 NPs. **d** COF between the NCGs and different materials. The NCGs were made from 25.0 mg mL^−1^ HAMA-28 NPs. **e** Change in COF of NCGs during a 6-h test at 25 °C. The NCGs were prepared from 25.0 mg mL^−1^ HAMA NPs with the DS of 15% (gray), 22% (orange), and 35% (blue). **f** Variation in COF of NCGs in four sequential 6-h friction tests. **g** Macroscopic and microscope images of NCGs before and after friction tests. The NCGs in (**f**, **g**) were prepared by 25.0 mg mL^−1^ HAMA-28 NPs. Error bars represent standard deviations based on three independent measurements

The lubrication properties of the NCGs were evaluated by using a rheometer at varying loads and shear rates. For the NCGs prepared from HAMA-28 NPs, at a constant shear rate of 0.1 s^−1^, the COF increased from 0.0019 ± 0.0005 to 0.0064 ± 0.0003 with the axial force increasing from 0.1 to 1.0 N, respectively (Fig. 5a). At a constant axial force of 0.3 N, the COF of the NCGs increased from 0.0022 ± 0.0003 to 0.0229 ± 0.0038 when the shear rate increased from 0.1 to 1.0 s^−1^, respectively (Fig. 5b). At the same axial force and shear rate, no significant changes in the COF of NCGs made from HAMA NPs with different DS were observed (Fig. 5a, b). Moreover, the COF of the NCGs increased from 0.0022 ± 0.0001 to 0.0053 ± 0.0002 as *C*_NPs_ increased from 8.3 to 133.3 mg mL^−1^ (Fig. 5c). For comparison, we also measured the COF of the hydrogels made from linear HAMA. The value of COF for these hydrogels increased from 0.04 to 0.09 with loads increasing from 0.3 to 1.0 N, respectively (Fig. S21), that is, a 15-fold increase took place, in comparison with the COF of the NCGs.

The reason for the lubrication originated from compression-indued squeezing of water from the NCG (Fig. S22 and Movie S1), which resulted in a hydration layer at the NCGs surface [57–59]. Moreover, hydrophilic dangling HA chains on the surface of HAMA NPs also present on the NCG surface (Figs. 1 and S6, Note S2). These chains further stabilized the hydration layer at the interface of NCGs and substrate [59–61], which lubricated the surfaces dramatically. The increase of *C*_NPs_ resulted in a smaller pore size and water content in the NCGs, possibly reducing the thickness of the hydration layer and thus leading to an increase in COF [62, 63].

Figure 5d shows that the values of COF upon sliding of the NCG against different materials were below 0.03, indicating excellent lubrication performance of the NCG. In particular, COF ≈ 0.0018 was achieved upon sliding the NCG against polyethylene, polypropylene, and polyurethane. The lubrication performance of the NCG was preserved in a 6 h friction test, with COF increasing only from ~ 0.005 to ~ 0.025 (Fig. 5e), although water could partly evaporate from the NCG in these experiments. No noticeable scratches and wear were observed on the hydrogel surface after 6-h friction test (Fig. S23). Moreover, after a 24-h friction test, the COF value for the NCGs increased only from 0.006 to 0.028 (Fig. 5f). Although several scratches were found on the NCG surface, no significant damage was observed (Fig. 5g). The COF of the NCGs was also evaluated upon hydrogel immersion in the simulated body fluid, resulting in a COF of 0.018 (Fig. S24). Such increase in COF was caused by the high ionic strength of the simulated body fluid, which resulted in NCG shrinking and reduction in water content (hence the decrease in the thickness of the hydration layer and a higher COF) [64]. The superior lubrication of NCGs can be further demonstrated through comparing their COF with those of lubricative hydrogels reported in the literature (Table S6).

### Hydrogel Biodegradation and Biocompatibility

**Fig. 6:**
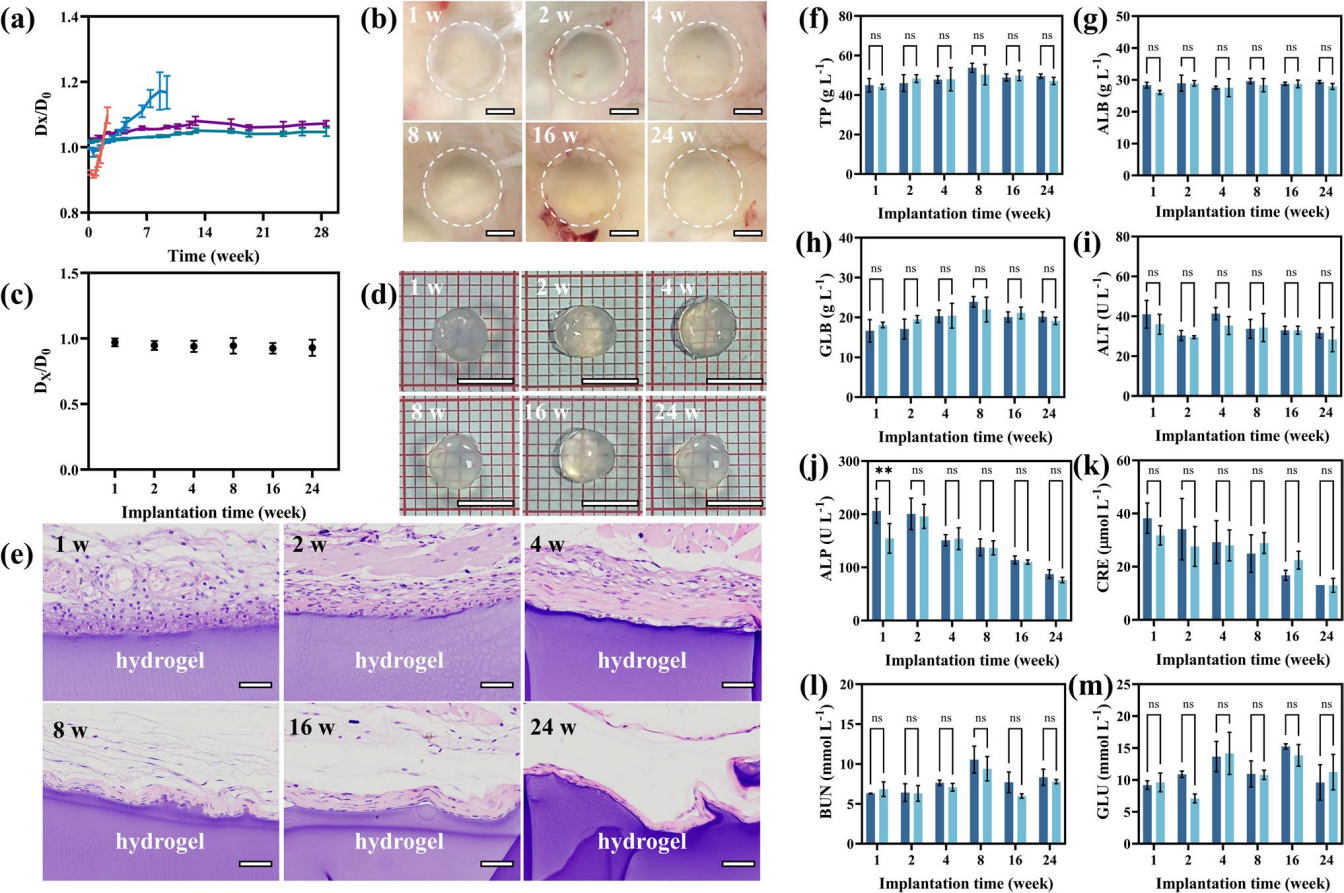
Degradation and biocompatibility of NCGs. **a** Degradation-driven diameter changes of NCG rods prepared by 25.0 mg mL^−1^ HAMA NPs with DS of 15% (orange), 22% (blue), 28% (purple), and 35% (green). D_x_ is the diameter of NCG rods at selected time points and D_0_ is the initial diameter of NCG rods. **b** Photographs of the mouse skin with the implanted NCG prepared from HAMA-28 NPs at 1, 2, 4, 8, 16, and 24 weeks of subcutaneous implantation. The scale bar is 0.25 cm. **c** The ratio of diameter changes after 1, 2, 4, 8, 16, and 24 weeks of subcutaneous implantation. D_x_ is the diameter of NCG discs at selected time points and D_0_ is the diameter of NCG discs before implantation. **d** Photographs of NCGs recovered from the implantation site of the skin. The scale bar is 0.5 cm. **e** H&E staining of the subcutaneous tissue with subcutaneous implantation of NCGs at 1, 2, 4, 8, 16, and 24 weeks. The scale bar is 50 μm. **f–m** Biochemical indices of liver and kidney of mice after subcutaneous implantation of NCGs in mice. Error bars represent standard deviations based on three independent measurements. Asterisks indicate statistically significant differences (^∗∗^*P* < 0.01) and “ns” indicate no statistically significant differences

Biodegradation of the NCGs was evaluated by measuring the change in hydrogel dimensions by immersing them in the hyaluronidase solution with the concentration of 200–500 units mL^−1^. The degradation of the NCGs was controlled by the DS of the constituent HAMA NPs. The NCG prepared from HAMA-15 NPs showed the fastest degradation rate, that is, the hydrogel completely degraded and dissolved in 3 weeks. The degradation of NCGs prepared by HAMA-22 NPs was completed after 9 weeks. Before their complete dissociation, the NCGs prepared from HAMA-15 and HAMA-22 NPs showed weak degradation-driven swelling [27], with ~ 10% and 20% increase in hydrogel dimensions, respectively. This degradation-driven swelling behavior was different from that of conventional hydrogels which can show up to three-fold increase of the initial dimension [27, 65]. Importantly, the NCGs prepared from HAMA-28 and HAMA-35 NPs showed no noticeable degradation within 28 weeks (Figs. 6a and S25).

The in vivo degradation of the NCGs was evaluated by using a subcutaneous implantation model in mice (Fig. 6b–d). Hydrogel discs with diameter and thickness of 4.7 and 1 mm, respectively, were implanted into the back of the Balb/C mice (Fig. S26a). Hydrogel degradation was evaluated by assessing the change in NCG diameter at selected time points. During 24 w of subcutaneous implantation, no macroscopic degradation and hydrolysis were observed by monitoring the diameter of the implanted NCG samples (Figs. 6b, c and S26b). Moreover, the implanted NCGs were easily recovered from the implanted location by surgery without any NCG damage (Fig. 6d). Such long-term resistance to biodegradation both in vitro and in vivo may be ascribed to the morphology of HAMA NPs which inhibited HA degradation by hyaluronidase [66].

To further evaluate the biocompatibility of the NCGs, their toxicity to mammalian cells was first examined. To promote cell adhesion, the NCG samples were prepared by copolymerizing 0.1% Arg-Gly-Asp (RGD) peptides modified by methacrylic acid (Fig. S27a) with HAMA NPs. No significant change in the mechanical properties of the hydrogel was observed after functionalization of the hydrogel with RGD (Fig. S27b). The viability of fibroblast L929 and hyaline chondrocyte cells was ~ 90% after 3-day cell culture (Figs. S28 and S29).

Next, the in vivo tissue response to NCGs was further evaluated. Figures 6e and S30 show representative histological sections of hematoxylin and eosin (H&E) stained subcutaneous tissues after implantation of the NCG discs for 24 weeks. The NCGs were covered with fibrous capsules that mainly consisted of fibroblasts and a few inflammatory cells such as eosinophils, neutrophils, macrophages, and lymphocytes. Despite the presence of the inflammatory cells, no calcification, abscess, necrosis, or tumorigenesis was observed at the implant site for up to 24 weeks. Furthermore, the histological grading system was used for the semi-quantitative evaluation of the histological response of the NCGs (Table S7) [50, 51]. The score of capsule quality increased from 2.83 ± 0.41 to 3.67 ± 0.52, the score of capsule thickness changed from 3.67 ± 0.52 to 4.33 ± 0.52, and the score of cell infiltration increased from 2.67 ± 0.52 to 3.50 ± 0.55 with the implantation time increasing from 1 to 24 weeks (Table S8). The results of histological grading also indicated that the number of inflammatory cells decreased at longer duration of subcutaneous implantation, which was consistent with the results of H&E staining.

Furthermore, the immunohistochemical analysis of iNOS, TNF-α, and IL-1β was conducted to evaluate local inflammatory responses over a 24-week period following the hydrogel implantation. At early time points (1–2 weeks), low-level positive staining for these markers was detected, indicating a mild acute inflammatory response (Fig. S31). The expression of all three cytokines exhibited a clear decrease from 4 to 8 weeks. At 16 and 24 weeks, the expression of inflammatory markers was minimal or nearly undetectable, indicating the absence of chronic inflammatory response (Fig. S32). Collectively, the NCGs provoked mild inflammatory response within 2 weeks, which reduced with the extension of implantation time, possibly, due to the host-initiated tissue repair [67] and the anti-inflammatory effect of HA [68]. Moreover, although minor degradation of the NCGs on the microscopic length scale occurred after 16-week subcutaneous implantation, only mild inflammatory responses was observed (Fig. S33).

The biosafety of the NCGs was further evaluated by hemolysis experiments and biochemical indices of the liver and kidney of mice with hydrogel implantation. The NCGs exhibited good compatibility with blood with their hemolysis rate being less than 1% (Fig. S34a). The supernatant of red blood cells treated with the NCGs was colorless and transparent (Fig. S34b). These results confirmed high compatibility of the NCG with blood. Moreover, no significant difference was observed in each index of liver and kidney function between the NCG groups and the control group during 24-w implantation (Fig. 6f–m), indicating that the NCG had no obvious toxicity in liver and kidney of mice. In addition, the heart, liver, spleen, lung, and kidney tissues were collected for histological analyses by H&E (Fig. S35). There was no histological abnormality in the NCG groups, in comparison with the control group during 24 weeks implantation, which further supported excellent biocompatibility of the NCGs in vivo.

## Conclusions

The engineered NCGs exhibited an important and highly impactful combination of properties which rendered the translational potential of NCGs. First, it is the nonswelling NCG behavior, originating from the existence of the nanoscopic hydrophobic domains in the HAMA NP interior, which were formed by the association of methacryloyl groups of the HAMA molecules. The nonswelling NCG properties were independent of the concentration and DS of HAMA NPs, as well as the temperature. Their nonswelling properties could enable a precise control over the release of therapeutics for prolonged or localized delivery in targeted tissues since the nonswelling could maintain the unchanged permeability of NCGs [7]. Their stability in physiological environments and ability to encapsulate drugs in hydrophobic regions would make NCG as a suitable platform for the advanced drug delivery. Secondly, it is a high NCG transparency and a broad range of the mechanical properties controlled by varying DS and the concentration of the constituent HAMA NPs. The tunable mechanical properties and biocompatibility of NCGs could make them as effective scaffolds for long-term cell culture and tissue engineering [32]. Third, the NCGs exhibited excellent lubricating and wear resistance properties, as well as long-term resistance to biodegradation both in vitro and in vivo. Their lubrication could reduce the frictional damage when NCGs would be used as hydrogel coatings on medical devices and bioelectronics [69].

While biopolymer hydrogels have been widely implemented in a broad range of biomedical applications and clinical settings [70], their applications for translational use are hindered by the relatively high solution viscosity, strong swelling, and uncontrolled degradation. The engineered NCG described in the present work overcomes these limitations. Furthermore, the NCGs in comparison with the hydrogels prepared from their linear HA analogue showed other advantages. For example, the dispersion of HAMA NPs had viscosity of only ~ 1% of that of HA solution, a linear biopolymer analog, with the same concentration. The low viscosity is helpful for maintaining the cell viability when the dispersion of HAMA NPs is used as bioinks for extrusion 3D printing [71–73]. Moreover, the homogenous and transparent hydrogels were achieved with up to ~ 30 folds of concentration changes, leading to 800-fold change in their shear moduli. Although HA hydrogels are extensively used in preclinical and clinical settings, they rapidly degrade by hyaluronidase with tissue half-lives ranging from hours to days [74]. Therefore, the development of HA-based hydrogels with long-term resistance to degradation is highly desirable. In the present work, HA NCGs exhibited long-term stability against hyaluronidase degradation for 6 months both in vitro and in vivo.

Importantly, the strategy for the preparation of NCGs with greatly enhanced properties is versatile and can be applied to other types of biopolymer colloidal hydrogels, e.g., gelatin or binary NCGs of gelatin and HA. Owing to their lubricating properties, and long-term degradation resistance they would be useful in long-term drug delivery, tissue engineering, soft-tissue augmentation, and regenerative medicine [70].

## Supplementary Information

Below is the link to the electronic supplementary material.

Supplementary file1 (DOCX 17297 KB)

Supplementary file2 (MP4 1320 KB)
