# Supplementary material for: Nonswelling Lubricative Nanocolloidal Hydrogel Resistant to Biodegradation
Source: Nanomicro Lett. 2025 Jul 11;17:327. doi: 10.1007/s40820-025-01830-0 (PMC12254464; doi:10.1007/s40820-025-01830-0)
Supplement: Supplementary file 1 — Supplementary file1 (DOCX 17297 KB) [file 40820_2025_1830_MOESM1_ESM.docx]

Supporting Information for

**Nonswelling Lubricative Nanocolloidal Hydrogel Resistant to Biodegradation**

Tiantian Ding^1a^, Chunxia Ren^2a^, Liyuan Meng^1^, Guoyong Han^1^, Yao Xue^1^, Wenlong Song^1^, Daowei Li^2^, Hongchen Sun^2^, Bai Yang^1^, and Yunfeng Li ^1,3^*

^1^State Key Laboratory of Supramolecular Structure and Materials, College of Chemistry, Jilin University, 2699 Qianjin Street, Changchun 130012, P. R. China

^2^Jilin Provincial Key Laboratory of Oral Biomedical Engineering, Hospital of Stomatology, Jilin University, Changchun, 130021, P. R. China

^3^Joint Laboratory of Opto-Functional Theranostics in Medicine and Chemistry, The First Hospital of Jilin University, Changchun, 130021, P. R. China

^a^ Tiantian Ding and Chunxia Ren contributed equally to this work.

*Corresponding author. E-mail: yflichem@jlu.edu.cn (Yunfeng Li)

**Note S1 SAXS data analysis**

The SAXS was conducted on an Xeuss 3.0 instrument equipped with Cu as radiation sources. The light tube power was 30 W and a wavelength of 1.54 Å was used as the X-ray radiation source with the beam area of 0.9 mm × 0.9 mm. The distance between samples to detector was 400 mm. Each SAXS pattern was collected within 60 min. The SAXS data were analyzed by Xenocs XSACT 2.7 software. The data in the range of 0.115-0.189 Å^-1^was fitted by the Guinier's laws [S1, S2],

I(q) = I(0)⋅exp($\text{-}\frac{\text{q}^{\text{2}}\text{R}_{\text{g}}^{\text{2}}}{\text{3}}$) (S1)

Where, I(q) is the scattering intensity at a given scattering vector q, I(0) is the scattering intensity at zero angle, R_g_ is the radius of gyration of the scattering particle, q is the scattering vector. The fitting leads to a radius of gyration (R_g_) of the nanophase separation. The radius of the nanophase separation is resulted from the Equation S2. The fitting results are listed in Table S3.

R =  $\sqrt{\frac{\text{5}}{\text{3}}}$ R_g_ (S2)

**Note S2 Discussion on dangling chains on the surface of HAMA NPs**

The existence of dangling chains on the surface of HAMA NPs was characterized by transverse (*T_2_*) relaxation times in NMR measurements (Fig. S6) [S3-S5]. The protons of methacryloyl groups were chosen for analysis by integrating the peak area in the software of Origin. The area of the NMR peak of methacryloyl motifs from HAMA NPs at each recovery time was fitted with a double-exponential decay to obtain the *T*_2_. The HAMA NPs showed two *T_2_* values for all the degree of substitution of methacryloyl groups on the HAMA NPs. The smaller *T_2_* value, ~ 20-30 ms, was derived from the methacryloyl groups on chains with lower mobility in the NP interior of the HAMA NPs. In the NP interior, the mobility of methacryloyl groups was confined by the crosslinked environment, leading to a shorter transverse relaxation time in NMR measurements. In contrast, the larger *T_2_* value, ~120-160 ms, was resulted from the polymer chains with higher mobility which are dangling on the surface of the HAMA NPs. In the preparation process of HAMA NPs by nanoprecipitation method, there were always polymer chains dangling on the surface of NPs, because the crosslinking degree of HAMA NPs by adipic dihydrazide was ~ 60%. In this case, methacryloyl groups of dangling polymer chains on the surface of the HAMA NPs show higher chain dynamics, leading to a larger transverse relaxation time in NMR measurements.

**Note S3 Crosslinking density of NCGs**

The crosslinking density (ρ_c_) of NCGs was calculated using Eq. S3 [S6],

$\text{ρ}\text{c}\text{ }\text{= }\frac{\text{1}}{\text{υ}\text{M}_{\text{c}}}$ (S3)

where *M_c_* is molecular weight between cross-links (g mol^-1^) and *υ* is the specific volume of polymer (i.e., the reciprocal of polymer density, *υ* = 0.59 cm^3^ g^-1^ for sodium hyaluronate [S7]).

The average molecular weight between cross-links, *M_c_* was calculated from the shear modulus according to the rubber elasticity theory [S8],

$\text{M}_{\text{c}}\text{ = }\frac{\text{RTc}}{\text{G}}$ (S4)

Where G is the shear modulus, c is polymer concentration (g m^-3^), R is the gas constant, and T is the temperature.

The shear modulus (G) was obtained by following equation [S9],

$\text{G = }\sqrt{{\text{G}\text{}}^{\text{2}}\text{ + }{\text{G}\text{}\text{}}^{\text{2}}}$ (S5)

G′ and G′′ represent the energy storage modulus and loss modulus obtained from the rheological test, respectively.

The calculated crosslinking density was listed in Table S4.

**Supplementary Figures**


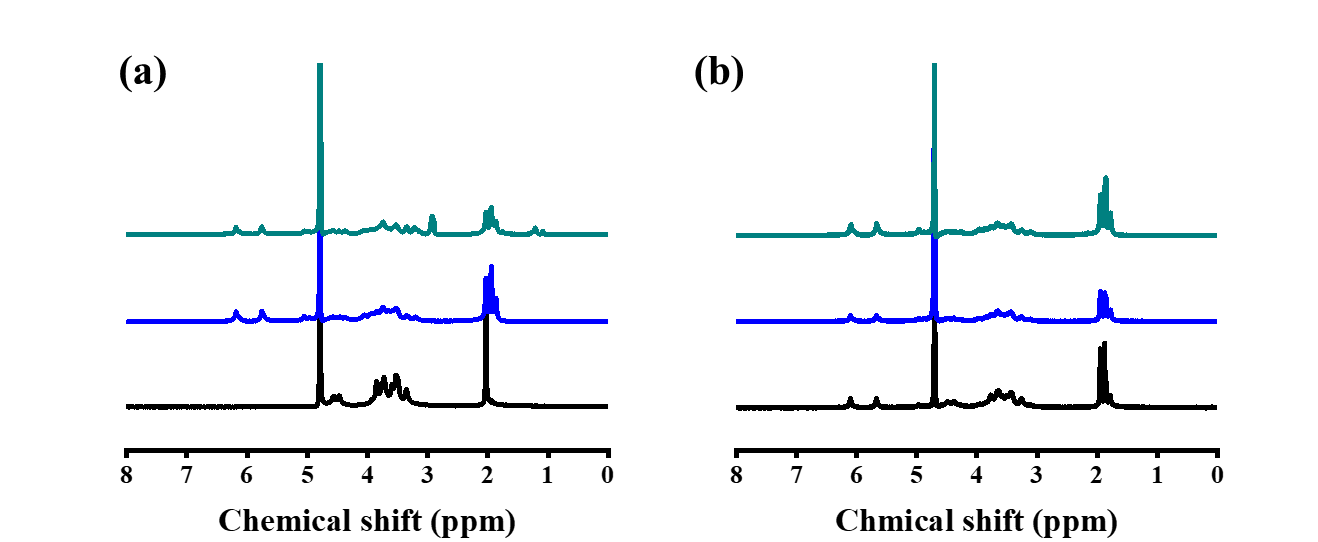


**Fig. S1** **^1^H** **nuclear magnetic spectrum (NMR) characterization of HA, HAMA, and HAMA NPs.** (**a**) ^1^H NMR of HA (black), HAMA-28 (blue), and HAMA-28 NPs (green). (**b**) ^1^H NMR of HAMA with DS of 15% (black), 22% (blue), and 35% (green)


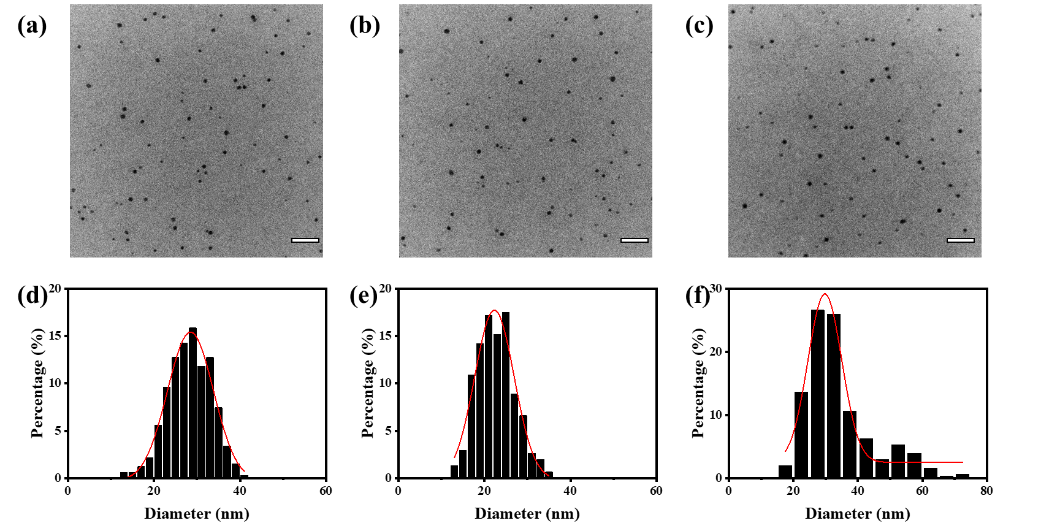


**Fig. S2 TEM characterization of HAMA NPs.** (**a-c**) TEM images of HAMA NPs with DS of 15% (**a**), 22% (**b**), and 35% (**c**). The scale bar is 200 nm. (**d-f**) The diameter distribution of HAMA NPs with DS of 15% (**d**), 22% (**e**), and 35% (**f**) by using software ImageJ to measure the size of HAMA NPs in TEM images


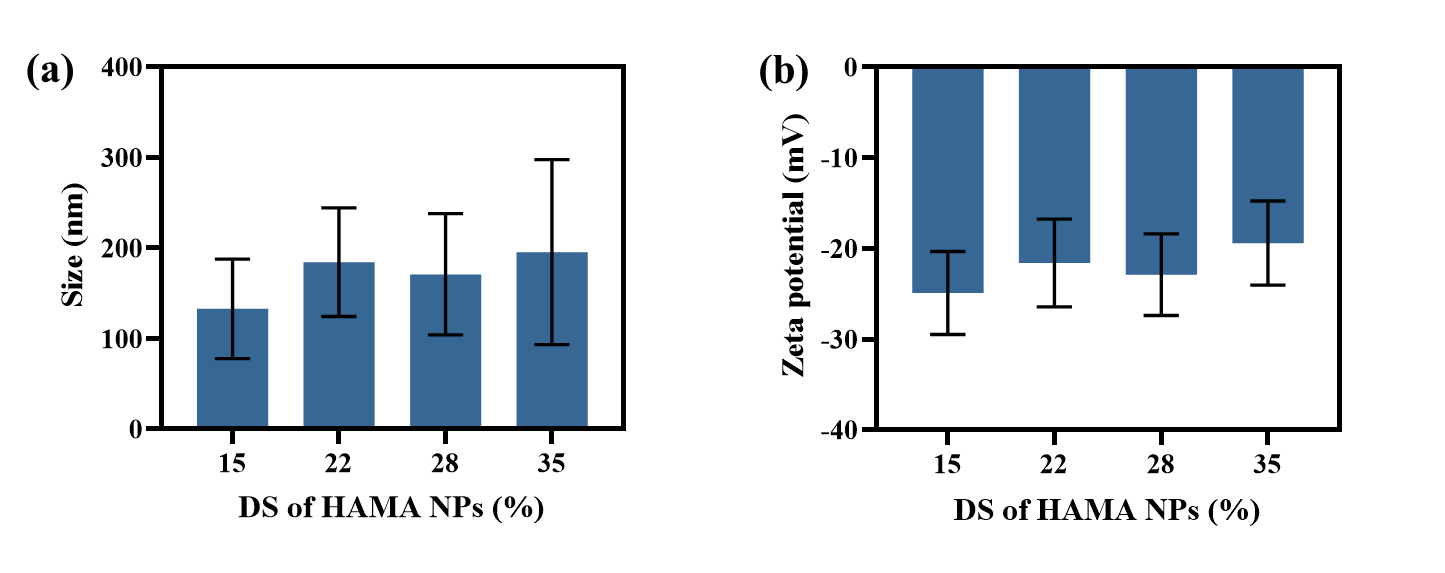


**Fig. S3 Size and zeta potential characterization of HAMA NPs.** (**a, b**) The hydrodynamic diameter (**a**) and the zeta potential (**b**) of HAMA NPs with different DS dispersed in deionized water. Error bars represent standard deviations based on three independent measurements


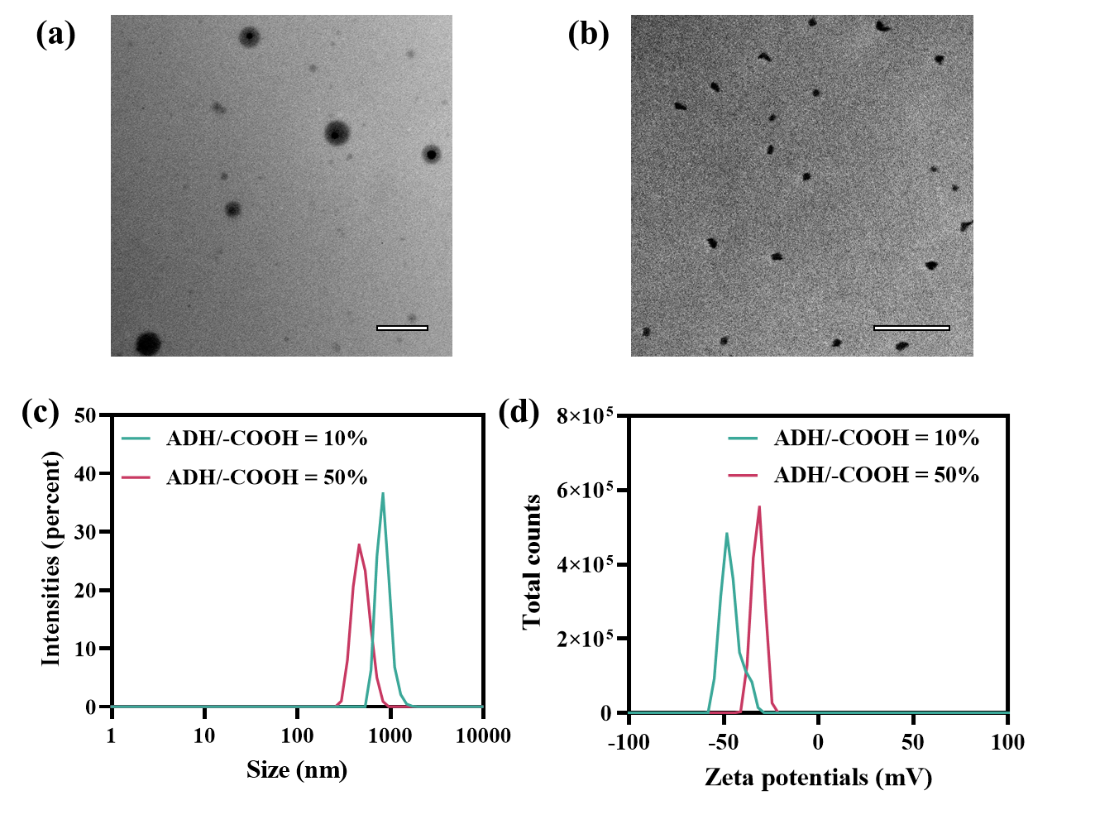


**Fig. S4** **Characterization of HAMA NPs with different dimensions.** (**a**) TEM image of HAMA NPs with the ratio between the added adipic dihydrazide (ADH) and the carboxyl groups (-COOH) of HAMA at 10%. The scale bar is 200 nm. (**b**) TEM image of HAMA NPs with the ratio between the added adipic dihydrazide (ADH) and the carboxyl groups (-COOH) of HAMA at 50%. The scale bar is 200 nm. (**c, d**) The hydrodynamic diameter (**c**) and zeta potential (**d**) of the HAMA NPs determined by DLS

**
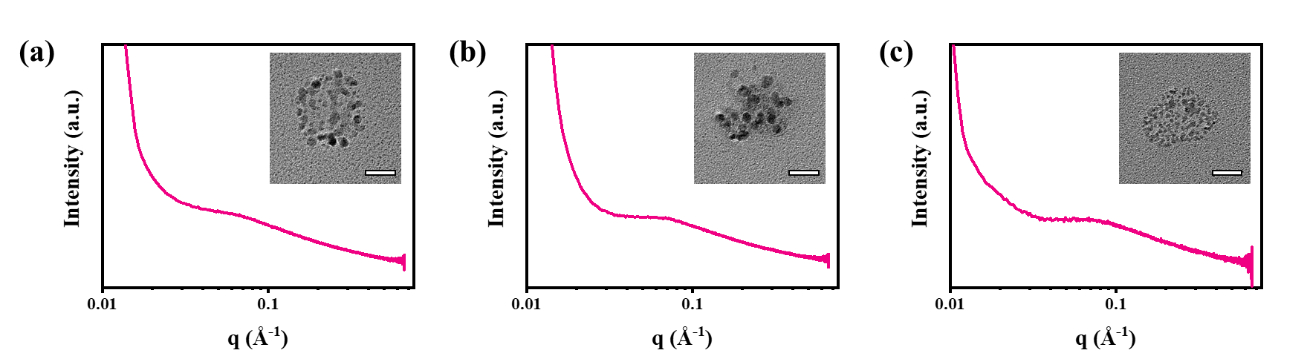
**

**Fig. S5 SAXS and TEM characterization of HAMA NPs.** (**a-c**) Experimental SAXS profiles of HAMA NPs in water with the DS of 15% (**a**), 22% (**b**), and 35% (**c**). Insets show the corresponding TEM images of HAMA NPs stained by ammonium tetrachloropalladate. The scale bar is 20 nm


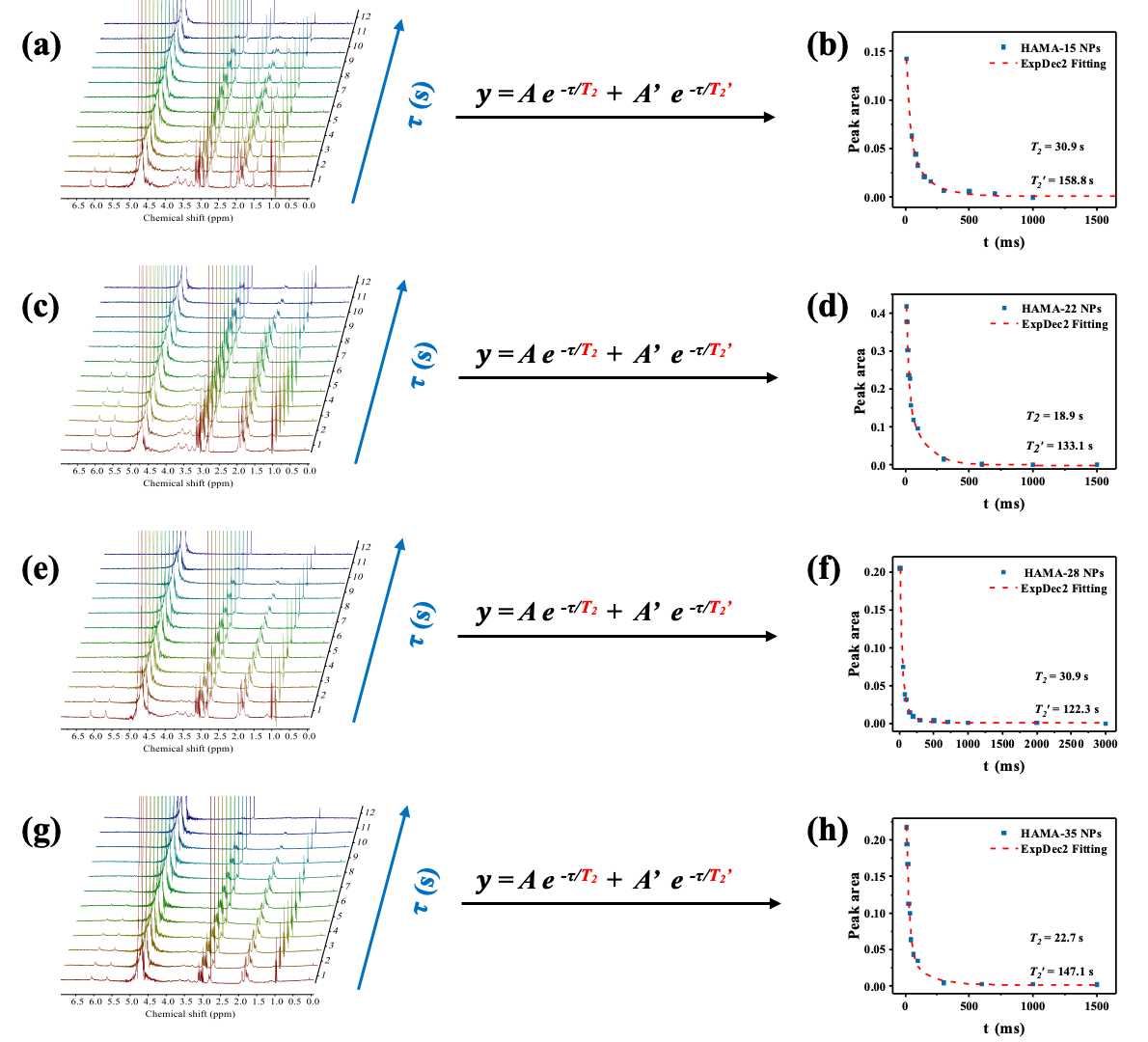


**Fig. S6 Transverse relaxation times (T_2_) in NMR measurements.** The HAMA NPs with the DS of 15% (**a**), 22% (**c**), 28% (**e**), and 35% (**g**) and corresponding fitting curves of the transverse relaxation peak area with respect to recovery time (τ). T_2_ values derived from the protons in double bonds of carbon in MA groups

**Fig. S7** **Viscosity characterization of HAMA and HAMA NPs.** Comparison of viscosity between 30.0 mg mL^-1^ HAMA-28 (green) and 30.0 mg mL^-1^ HAMA-28 NPs (pink). Error bars represent standard deviations based on three independent measurements

**Fig. S8** **Transmission** **characterization of NCGs.** The transmission of NCGs prepared with different DS of HAMA NPs

**
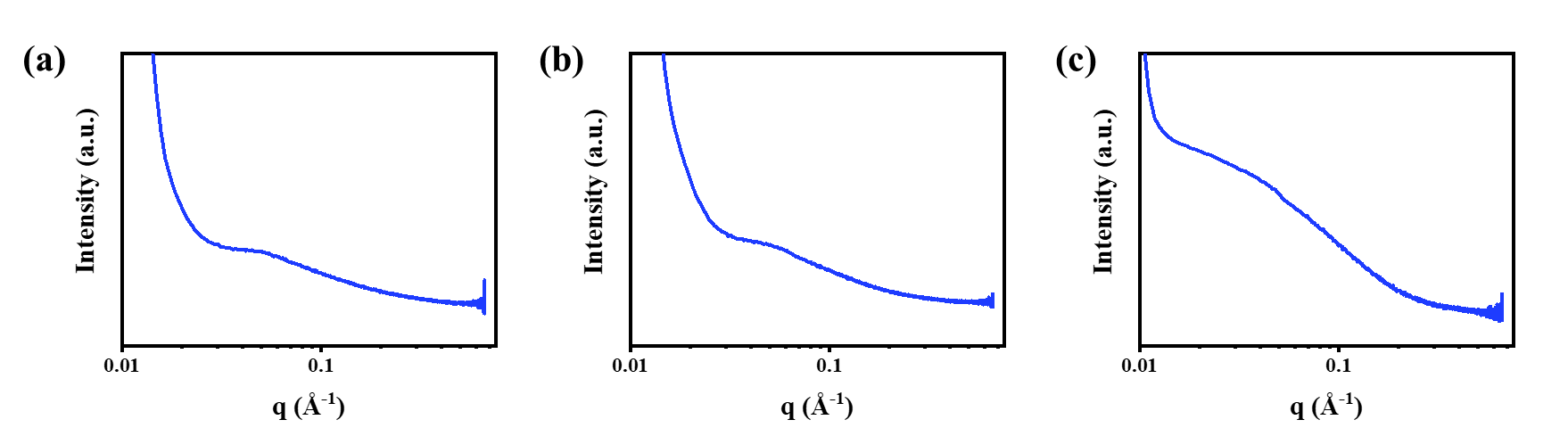
**

**Fig. S9** **SAXS characterization of NCGs.** (**a-c**) Experimental SAXS profiles of NCGs made from 25.0 mg mL^-1^ HAMA NPs with the DS of 15% (**a**), 22% (**b**), and 35% (**c**)


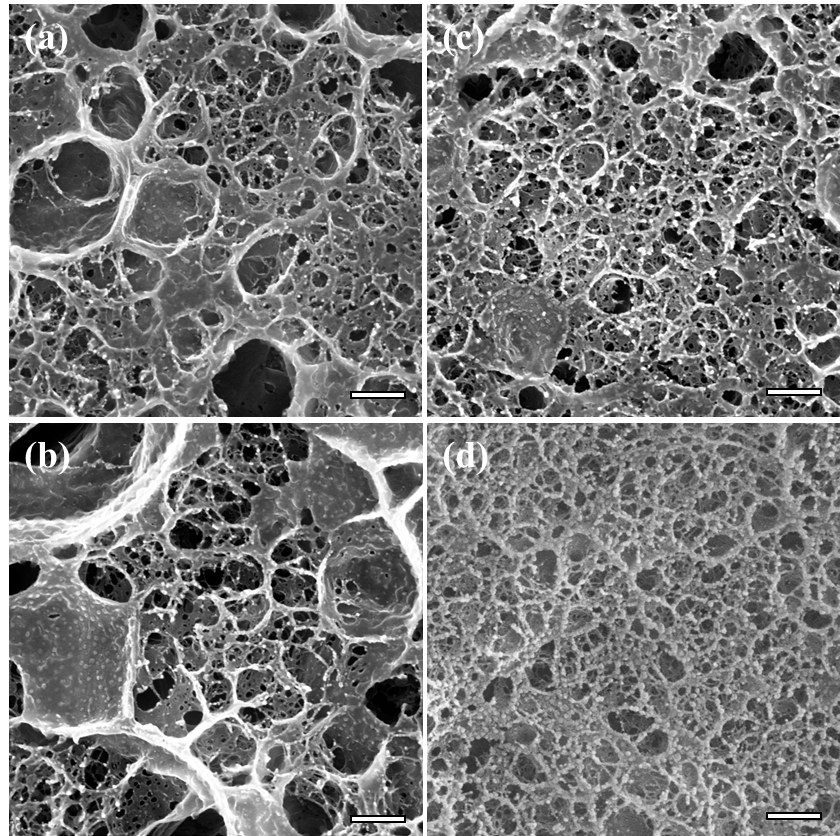


**Fig. S10** **Cryo-SEM characterization of NCGs.** Cryo-SEM images of NCGs prepared from 25.0 mg mL^-1^ HAMA NPs with DS of 15% (**a**), 22% (**b**), 28% (**c**), and 35% (**d**), respectively. The scale bar is 500 nm


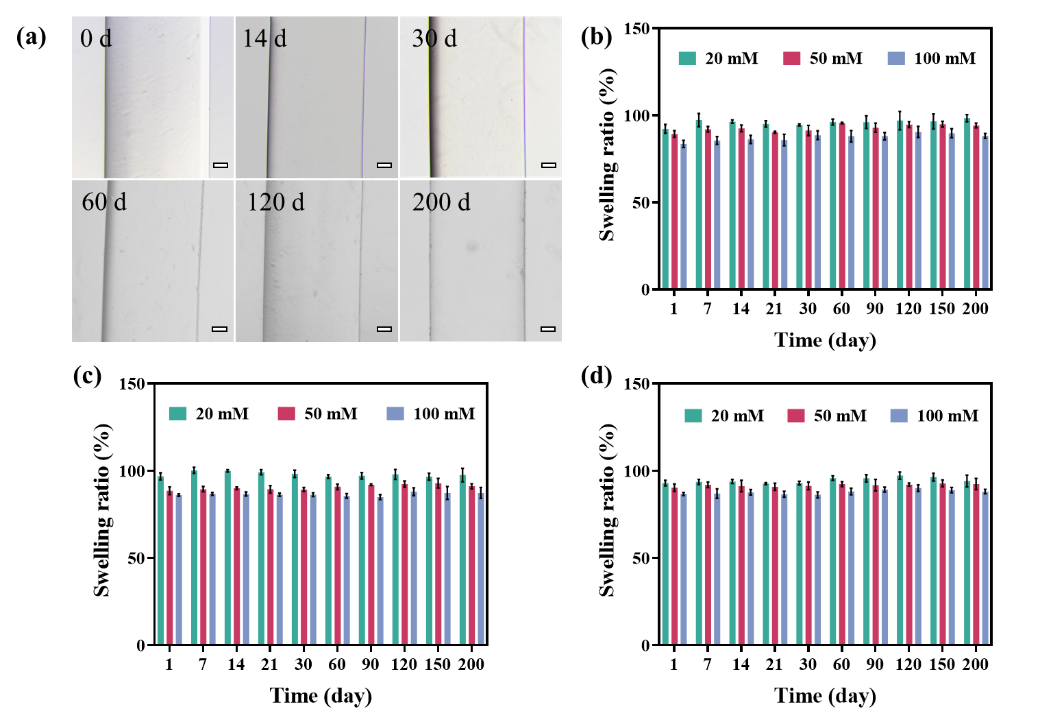


**Fig. S11** **Volume swelling of NCGs immersed in PBS.** (**a**) The representative optical microscope images of the NCGs made from 25.0 mg mL^-1^ HAMA-15 NPs after being immersed in 100.0 mM PBS for different durations at 25 °C. The scale bar is 100 μm. (**b-d**) Volume swelling ratio of NCGs made from 25.0 mg mL^-1^ HAMA-15 NPs (**b**), HAMA-22 NPs (**c**), and HAMA-35 NPs (**d**) after being immersed in PBS with 20.0, 50.0, and 100.0 mM PBS for different durations at 25 °C. Error bars represent standard deviations based on three independent measurements


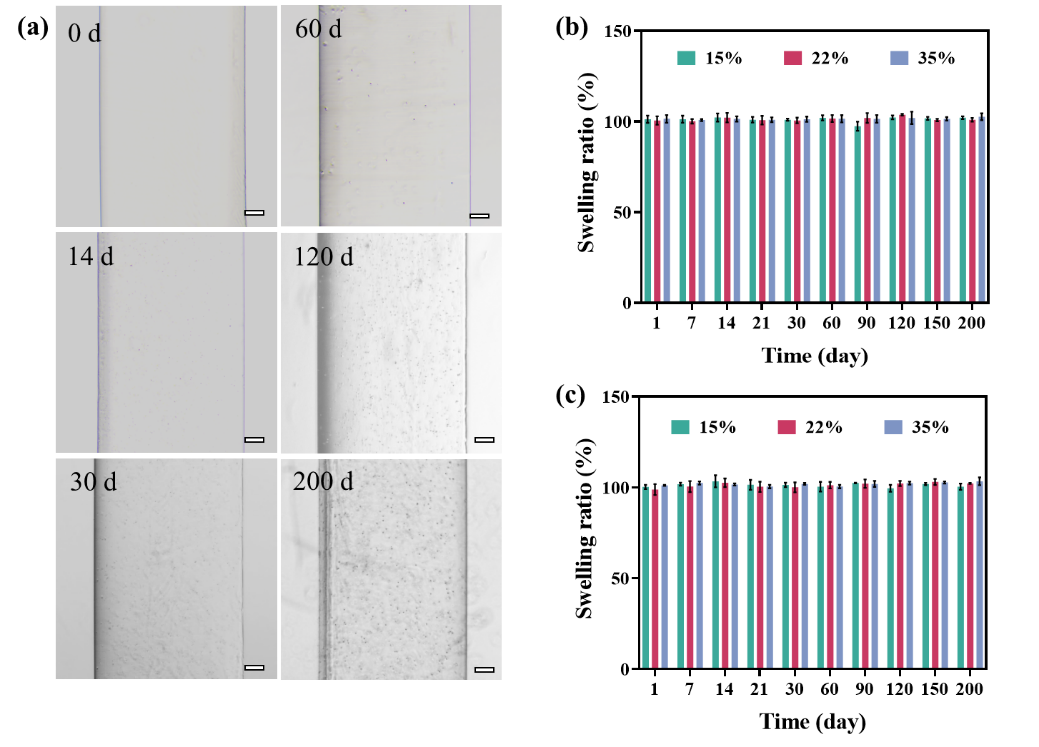


**Fig. S12** **Volume swelling of NCGs at different temperatures.** (**a**) The representative optical microscope images of the NCGs made from 25.0 mg mL^-1^ HAMA-15 NPs after being immersed in PBS solution (10.0 mM, pH = 7.4) for different durations at 25 °C. The scale bar is 100 μm. (**b, c**) Volume swelling ratio of NCGs prepared from 25.0 mg mL^-1^ HAMA NPs with different DS at 4 °C (**b**) and 25 °C (**c**). Error bars represent standard deviations based on three independent measurements


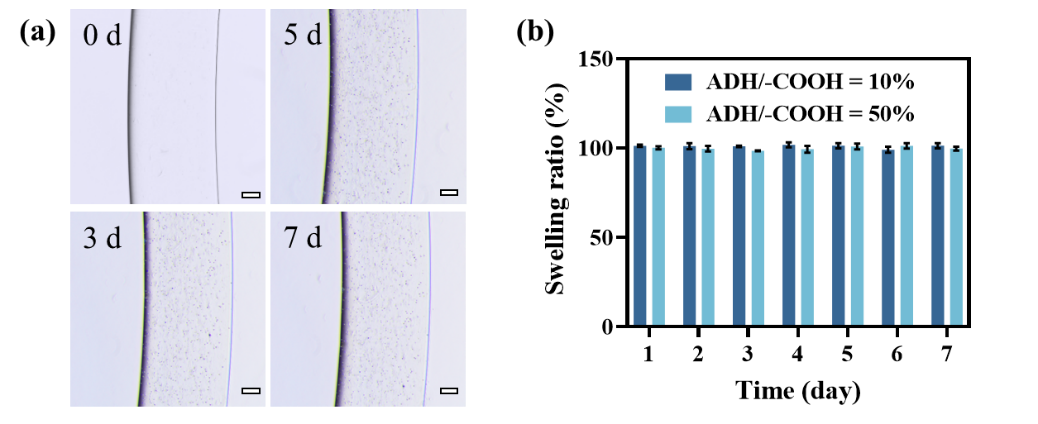


**Fig. S13 Volume swelling of NCGs prepared by HAMA NPs with different dimensions.** (**a**) The representative optical microscope images of the NCGs made from 25.0 mg mL^-1^ HAMA-28 NPs with the ratio between the added adipic dihydrazide (ADH) and the carboxyl groups (-COOH) of HAMA at 50% after being immersed in PBS (10.0 mM, pH = 7.4) solution for 7 d at 25 °C. The scale bar is 100 μm. (**b**) Volume swelling ratio of NCGs prepared from 25.0 mg mL^-1^ HAMA NPs with different feeding ratios of added adipic dihydrazide (ADH) and the carboxyl groups (-COOH) of HAMA after being immersed in PBS (10.0 mM, pH = 7.4) solution for 7 d at 25 °C. Error bars represent standard deviations based on three independent measurements


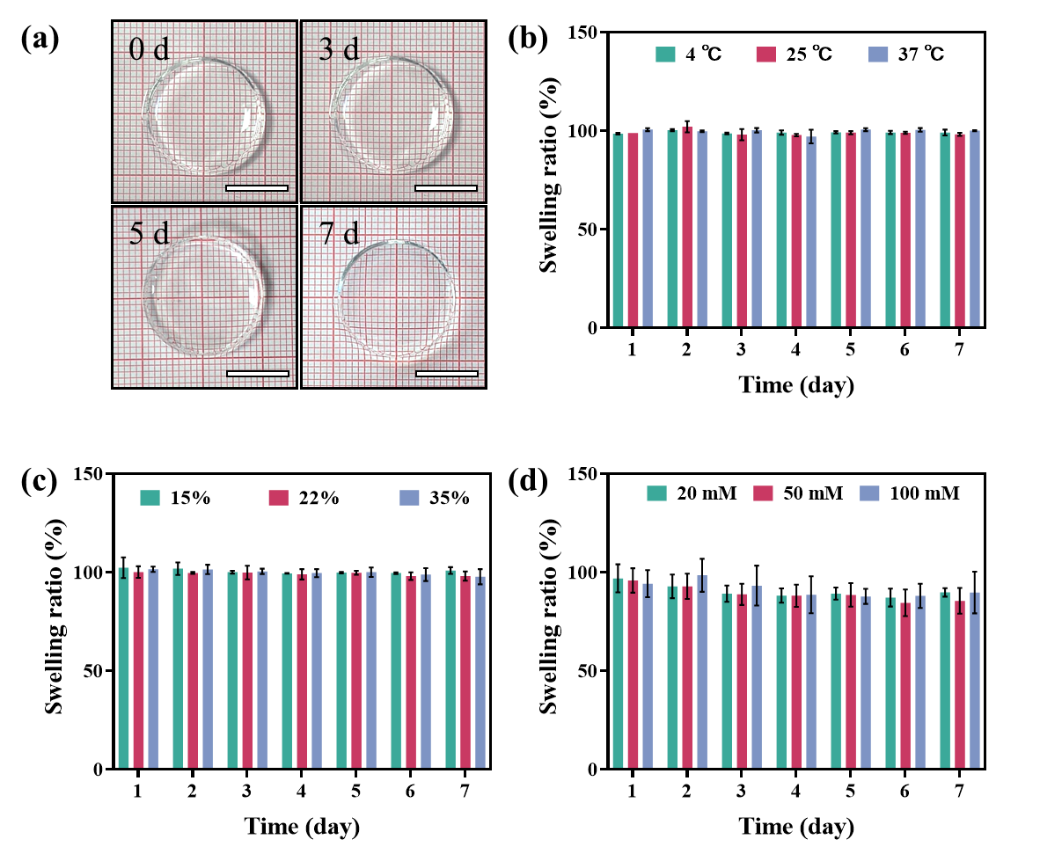


**Fig. S14 Mass swelling of NCGs.** (**a**) The representative photographs of NCGs made from 25.0 mg mL^-1^ HAMA-28 NPs after being immersed in PBS (10.0 mM, pH = 7.4) solution for 7 d at 25 °C. The scale bar is 1 cm. (**b**) Mass swelling ratio of NCGs made from 25.0 mg mL^-1^ HAMA-28 NPs at 4, 25, and 37 °C. (**c**) Mass swelling ratio of NCGs made from 25.0 mg mL^-1^ HAMA NPs with the DS of 15%, 22%, and 35% at 25 °C. (**d**) Mass swelling ratio of NCGs made from 25.0 mg mL^-1^ HAMA-28 NPs in PBS with 20.0, 50.0, and 100.0 mM at 25 °C. Error bars represent standard deviations based on three independent measurements


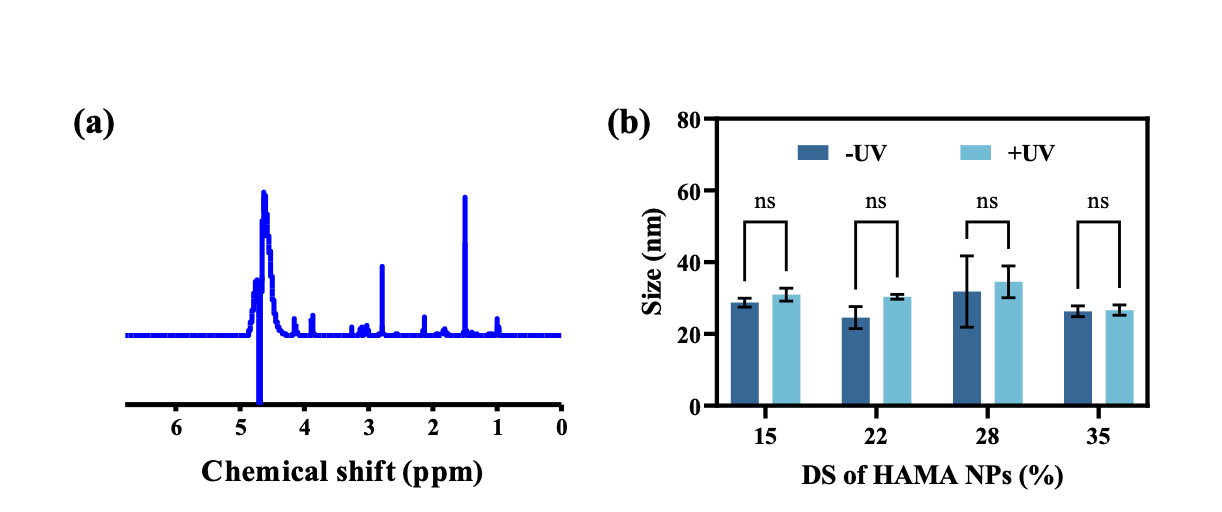


**Fig. S15 Characterization of HAMA NPs before and after photocrosslinking.** (**a**) ^1^H NMR of HAMA-28 NPs after 3-min photocrosslinking showing the peak disappearance of methacryloyl motifs. (**b**) The hydrodynamic diameter changes of HAMA NPs dispersed in PBS before (dark blue) and after UV (light blue) photocrosslinking. Error bars represent standard deviations based on three independent measurements. The “ns” represents no statistically significant differences. A dispersion containing 0.5 mg mL^-1^ HAMA NPs and 0.05% Irgacure 2959 were treated by UV light irradiation (Uvitron, 365 nm, 10 mW cm^-2^) for 3 min before DLS and NMR measurement


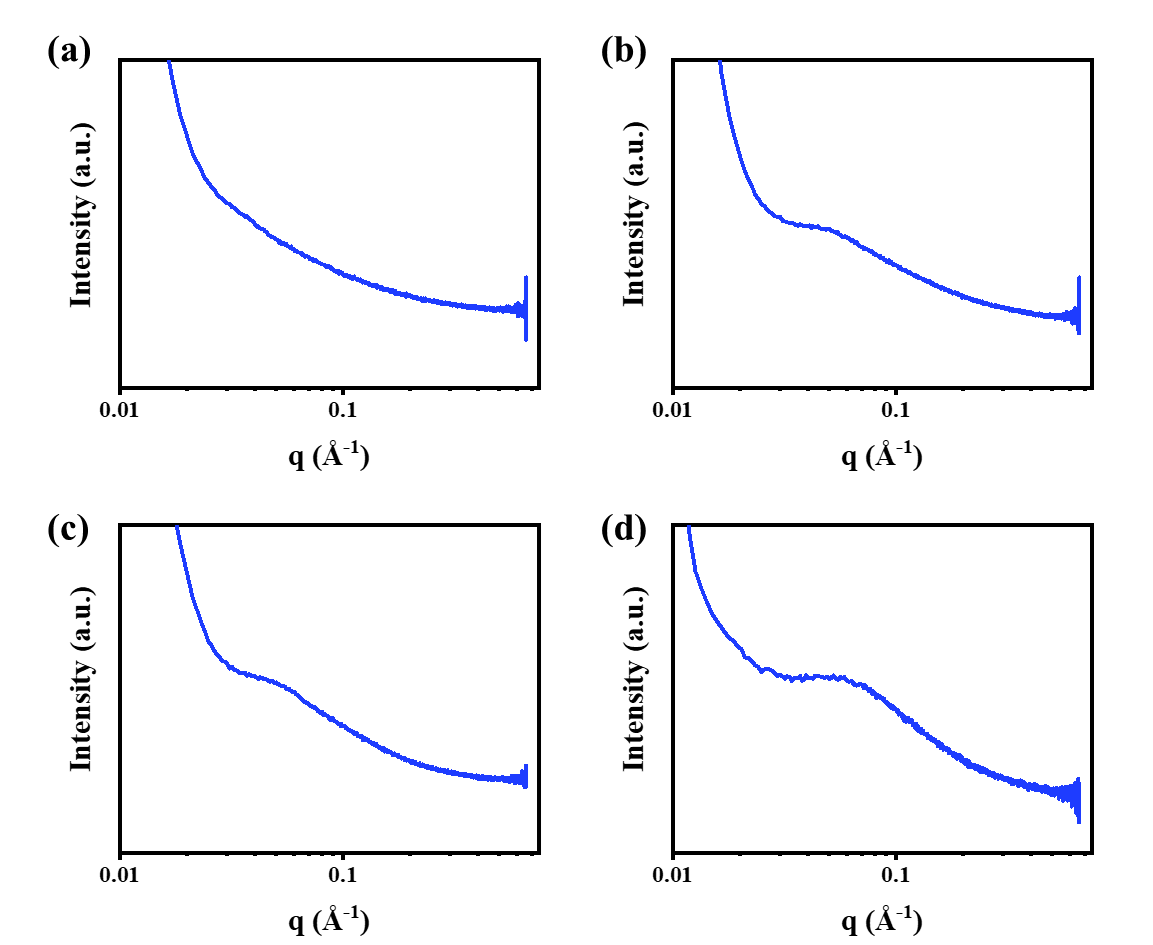


**Fig. S16 SAXS profiles of HAMA NPs after UV crosslinking.** (**a-d**) Experimental SAXS profiles of HAMA NPs with the DS of 15% (**a**), 22% (**b**), 28% (**c**) and 35% (**d**) after UV crosslinking. The *C*_NPs_ is 0.5 mg mL^-1^


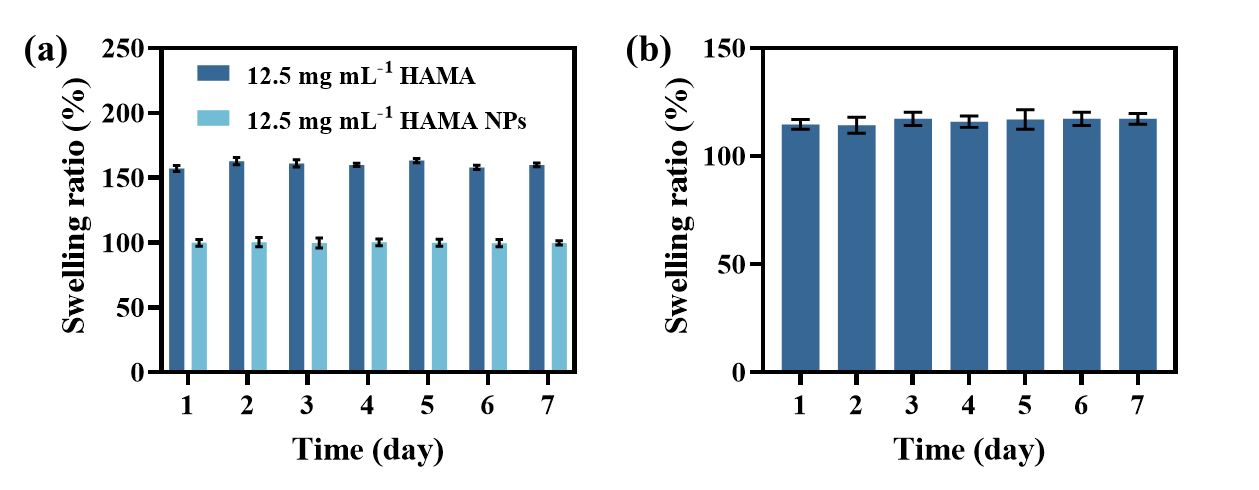


**Fig. S17 The swelling ratio of different hydrogels.** (**a**) Volume swelling of NCGs and hydrogel made from linear HAMA. The concentration of HAMA-28 NPs and HAMA-28 in the hydrogels are 12.5 mg mL^-1^. (**b**) Mass swelling of colloidal hydrogel prepared from HA NPs modified by aldehyde-group and gelatin NPs. The final concentrations of HA NPs modified by aldehyde-group and gelatin NPs are 13.3 and 80.0 mg mL^-1^, respectively. Error bars represent standard deviations based on three independent measurements


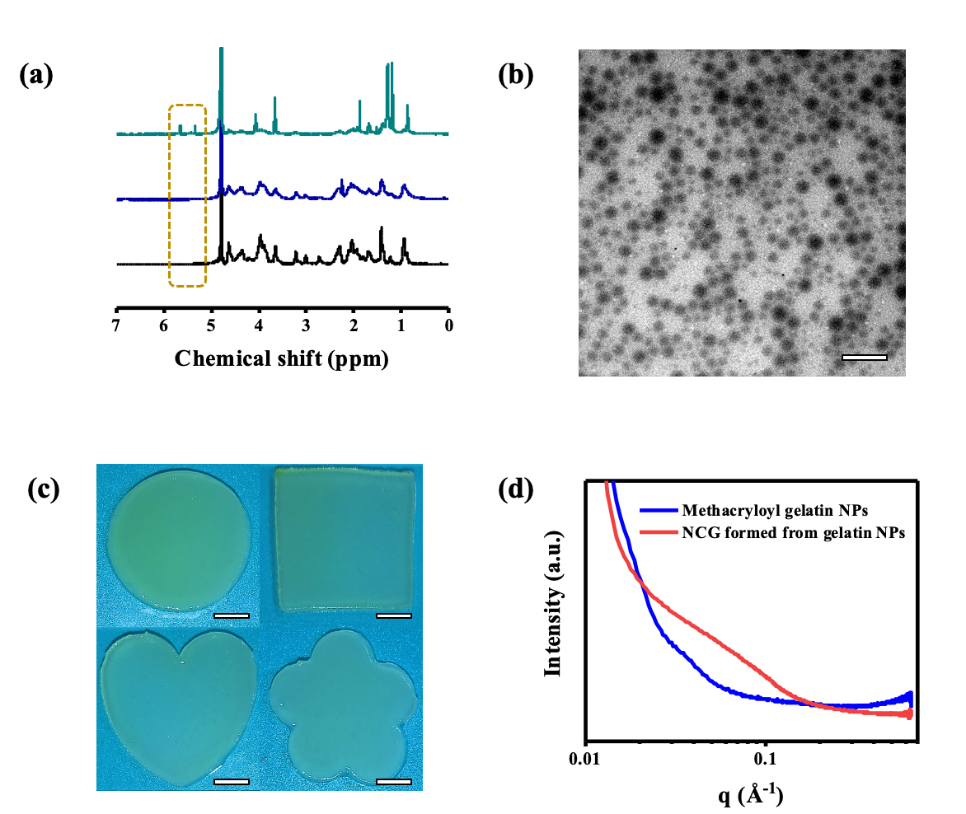


**Fig. S18** **Characterization of NCGs made from methacryloyl gelatin NPs.** (**a**) ^1^H NMR characterization of gelatin (black), gelatin NPs (blue), and methacryloyl gelatin NPs (green). (**b**) TEM image of methacryloyl gelatin NPs. The scale bar is 500 nm. (**c**) Macroscopic images of NCGs with different shapes prepared from 150.0 mg mL^-1^ methacryloyl gelatin NPs. The scale bar is 0.5 cm. (**d**) SAXS profile of methacryloyl gelatin NPs in water and NCG made from 50.0 mg mL^-1^ methacryloyl gelatin NPs


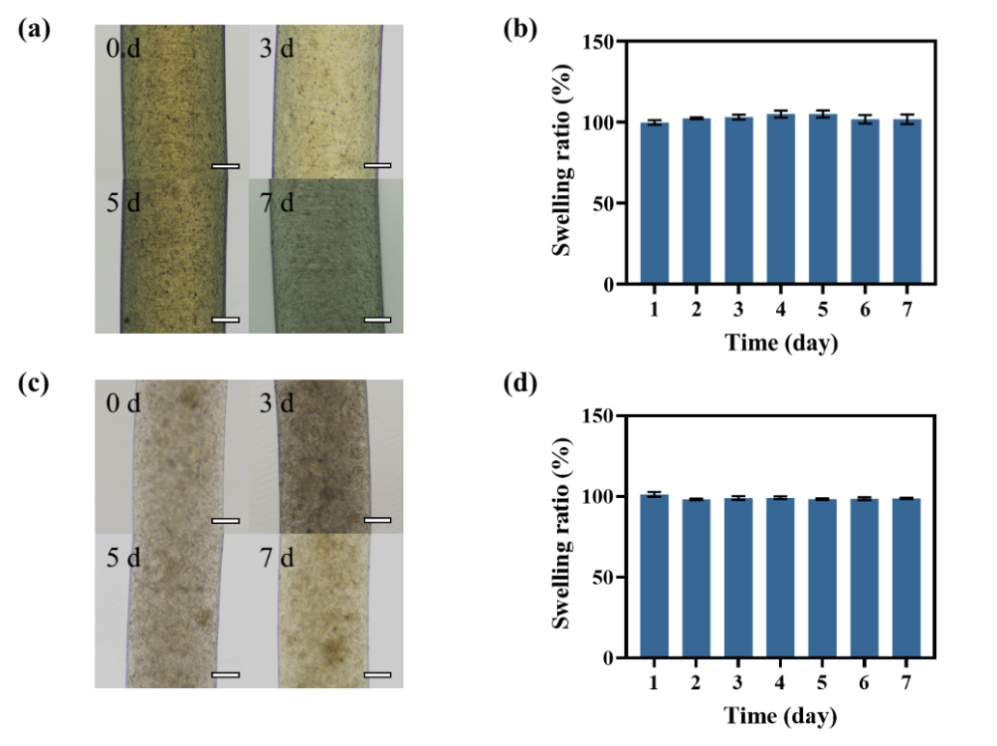


**Fig. S19 The nonswelling properties of NCGs**. (**a, b**) The representative optical microscope images (**a**) and volume swelling ratio (**b**) of NCGs made from 150.0 mg mL^-1^ methacryloyl gelatin NPs after being immersed in PBS (10.0 mM, pH = 7.4) for 7 d at 25 ℃. (**c, d**) The representative optical microscope images (**c**) and volume swelling ratio (**d**) of NCGs made from 20.0 mg mL^-1^ HAMA-28 NPs and 16.7 mg mL^-1^ methacryloyl gelatin NPs after being immersed in PBS (10.0 mM, pH = 7.4) for 7 d at 25 ℃. Error bars represent standard deviations based on three independent measurements


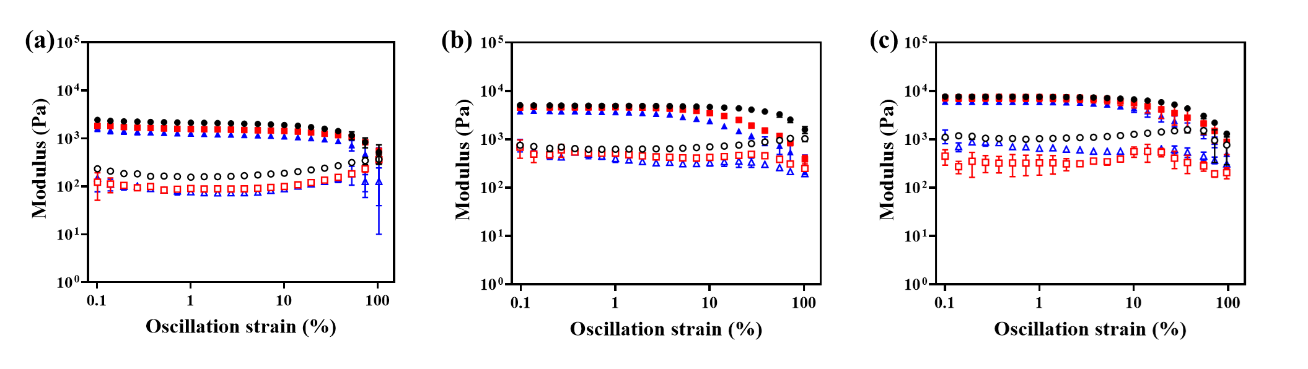


**Fig. S20** **Mechanical properties of NCGs**. Storage modulus, G′ (solid symbols), and loss modulus, G′′ (open symbols) changes of NCGs prepared with 25.0 mg mL^-1^ HAMA NPs with DS of 15% (**a**), 22% (**b**), and 35% (**c**) immersed in PBS for 7 (red), 15 (blue), and 30 d (pink). Error bars represent standard deviations based on three independent measurements


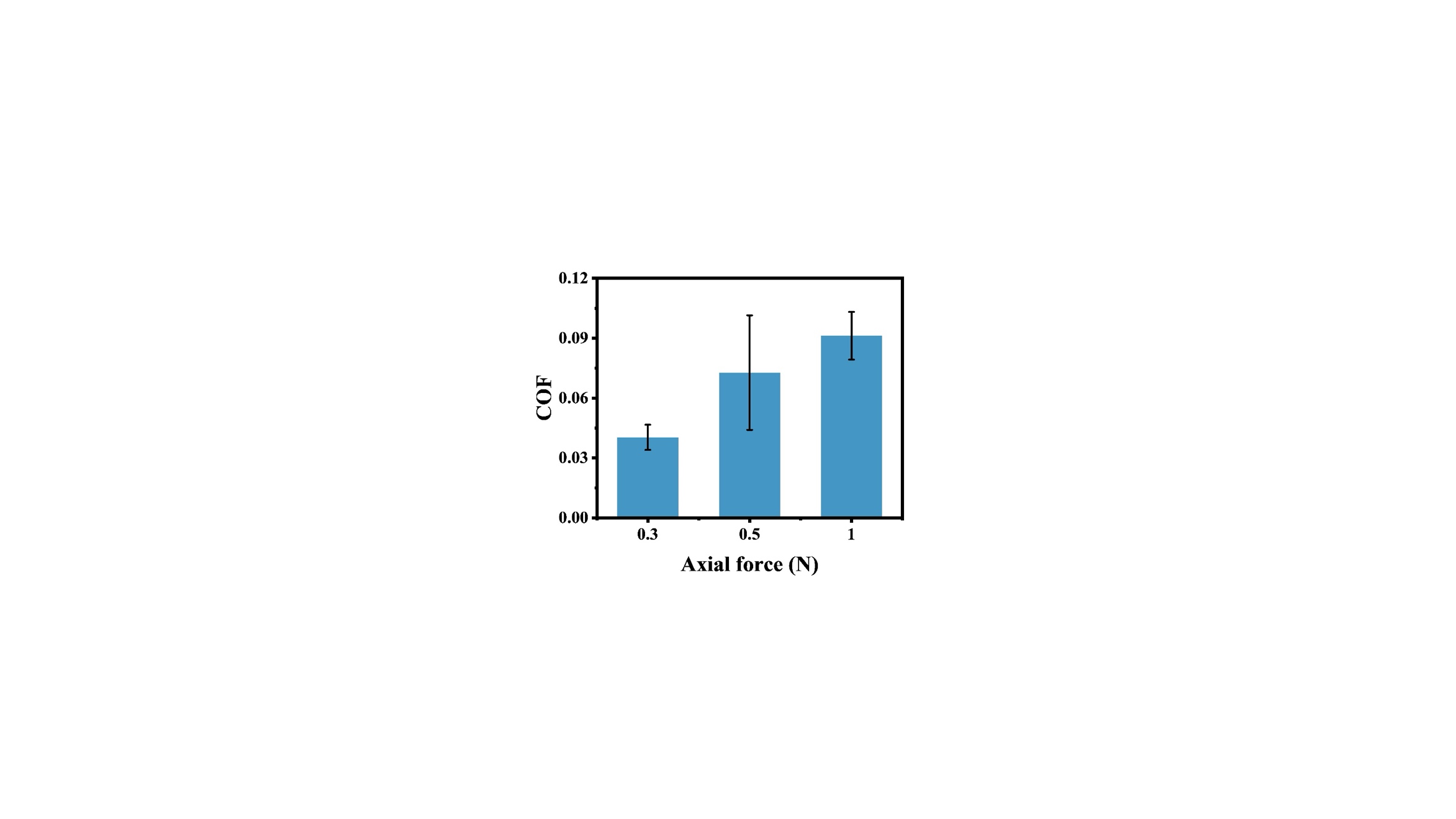


**Fig. S21 Lubricating properties of HAMA hydrogel.** The COF of HAMA hydrogel measured at 25 ℃ under axial forces in the range of 0.3 to 1.0 N at the constant shear rate of 0.1 s^-1^. The concentration of HAMA-28 in the hydrogel was 25 mg mL^-1^. Error bars represent standard deviations based on three independent measurements

**
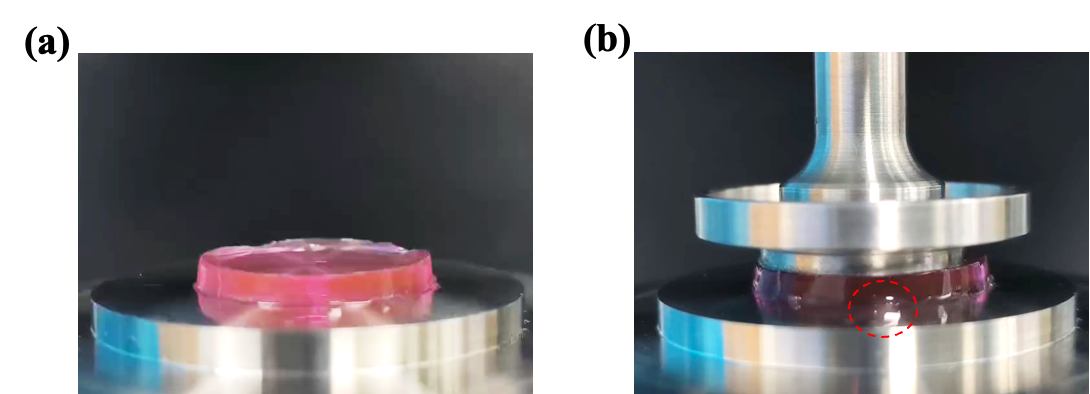
**

**Fig. S22 Characterization of water squeezed out from NCG during the friction test.** Photographs of NCG before friction test (**a**) and in the process of axial force loading (**b**). The red circle illustrates the water squeezed out from NCG


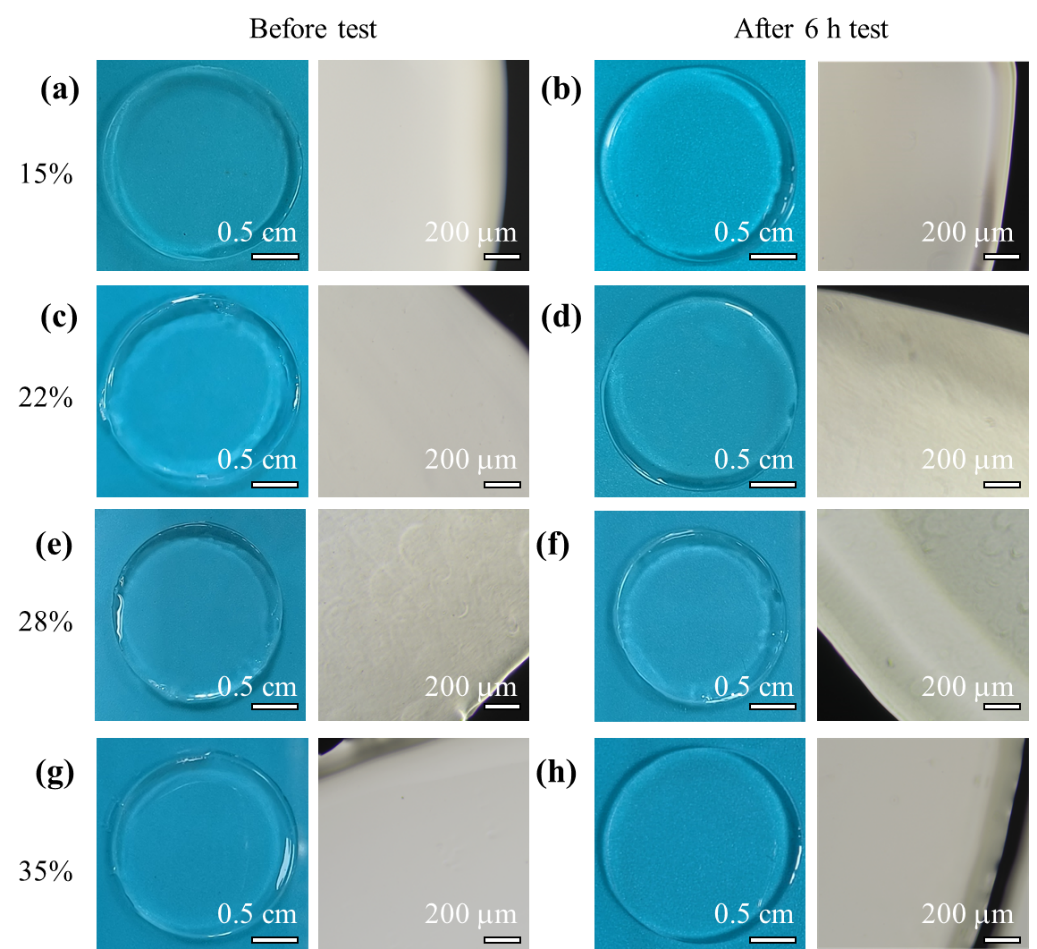


**Fig. S23 Macroscopic and microscopic images of NCGs before and after friction tests.** (**a-h**) The photographs and microscope images of NCGs before and after 6 h friction tests


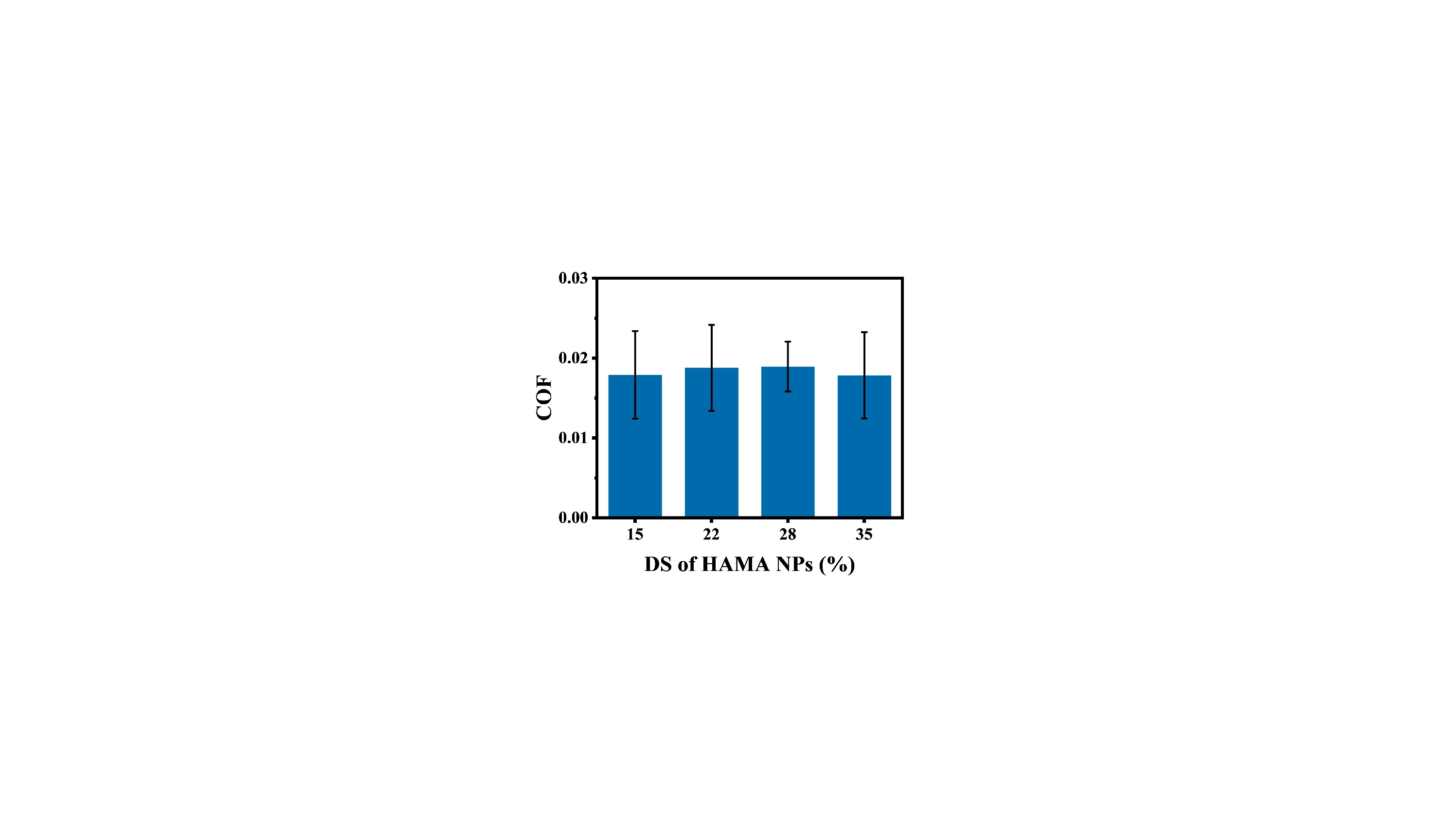


**Fig. S24 Lubricating properties of NCGs in simulated body fluid.** The COF of NCGs measured at 25 ℃ under axial forces of 0.3 N and the constant shear rate of 0.1 s^-1^. The measurement was performed in the simulated body fluid. Error bars represent standard deviations based on three independent measurements


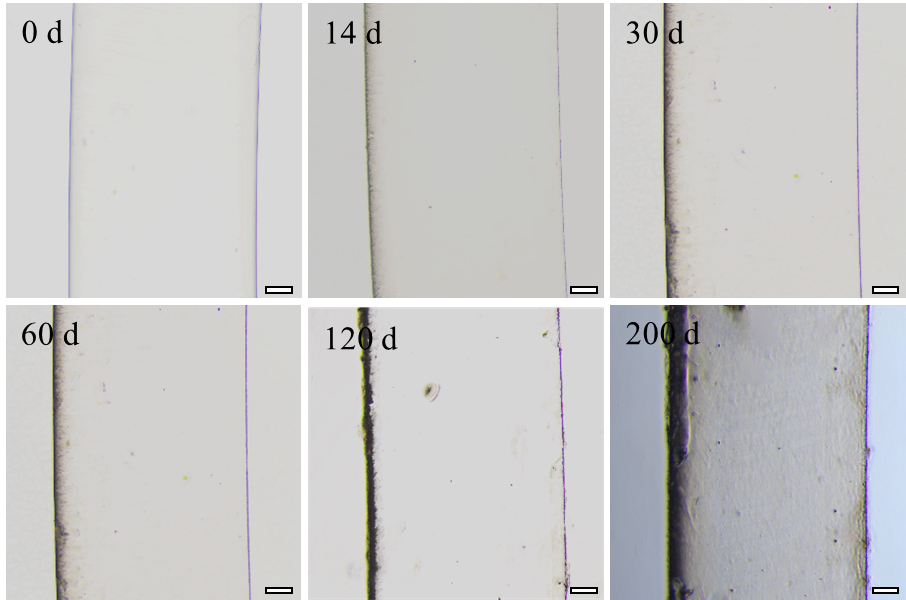


**Fig. S25 In vitro degradation of NCGs.** The representative optical microscope images of the NCGs made from 25.0 mg mL^-1^ HAMA-28 NPs after being immersed in PBS solution containing 200-500 units mL^-1^ hyaluronidase for 200 d at 37 °C. The scale bar is 100 μm


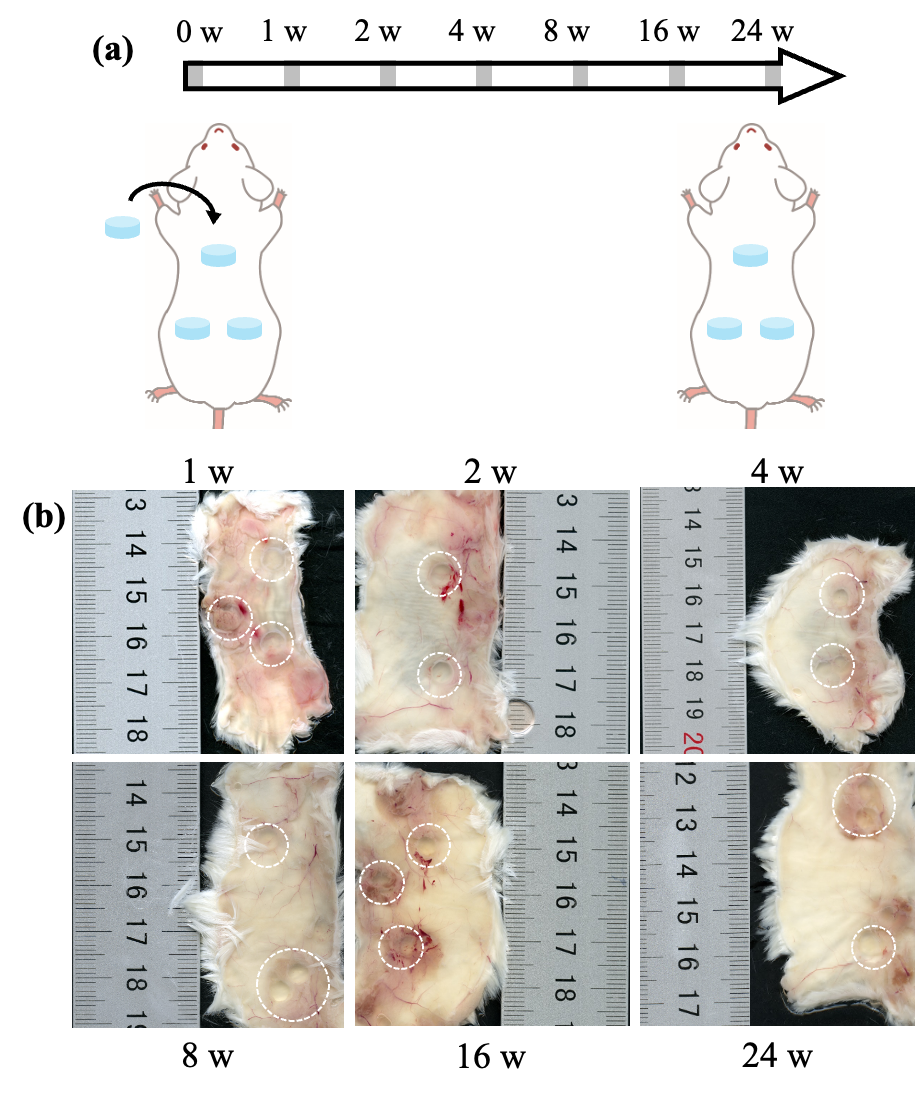


**Fig. S26** **Subcutaneous implantation of NCGs.** (**a**) Schematic illustration of NCGs implanted subcutaneously into the back of a mouse. (**b**) The photographs of the mice skin with the implanted NCGs at 1, 2, 4, 8, 16, and 24 w of subcutaneous implantation


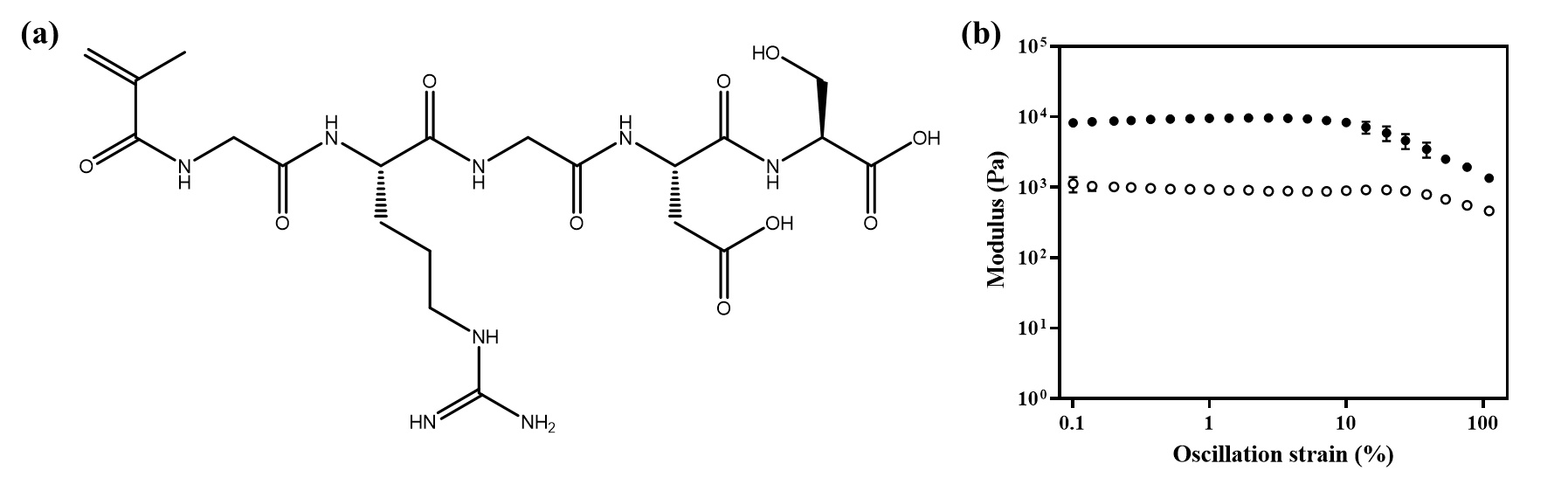


**Fig. S27** **The characterization of NCGs with Arg-Gly-Asp (RGD) peptides.** (**a**) Structural formula of RGD peptides modified with methacryloyl group. (**b**) Storage modulus, G′ (solid symbols), and loss modulus, G′′ (open symbols) of NCGs prepared by copolymerization of 0.1% RGD modified with methacryloyl group and 25.0 mg mL^-1^ HAMA-28 NPs. Error bars represent standard deviations based on three independent measurements


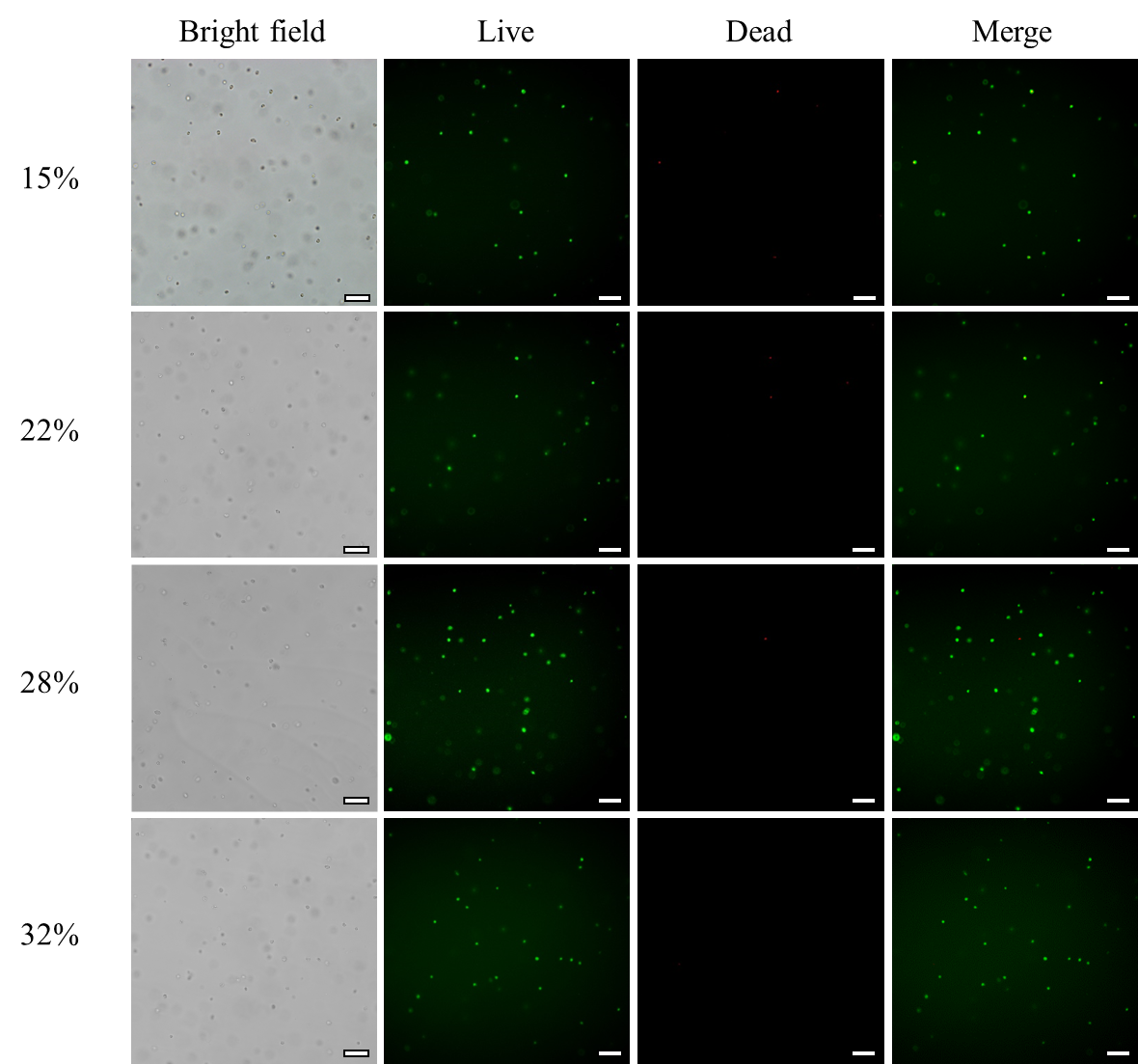


**Fig. S28** **Cell biocompatibility of NCGs.** Live/dead staining of L929 cells after three-dimensional (3D) culture in NCGs for 3 d. The scale bar is 100 μm


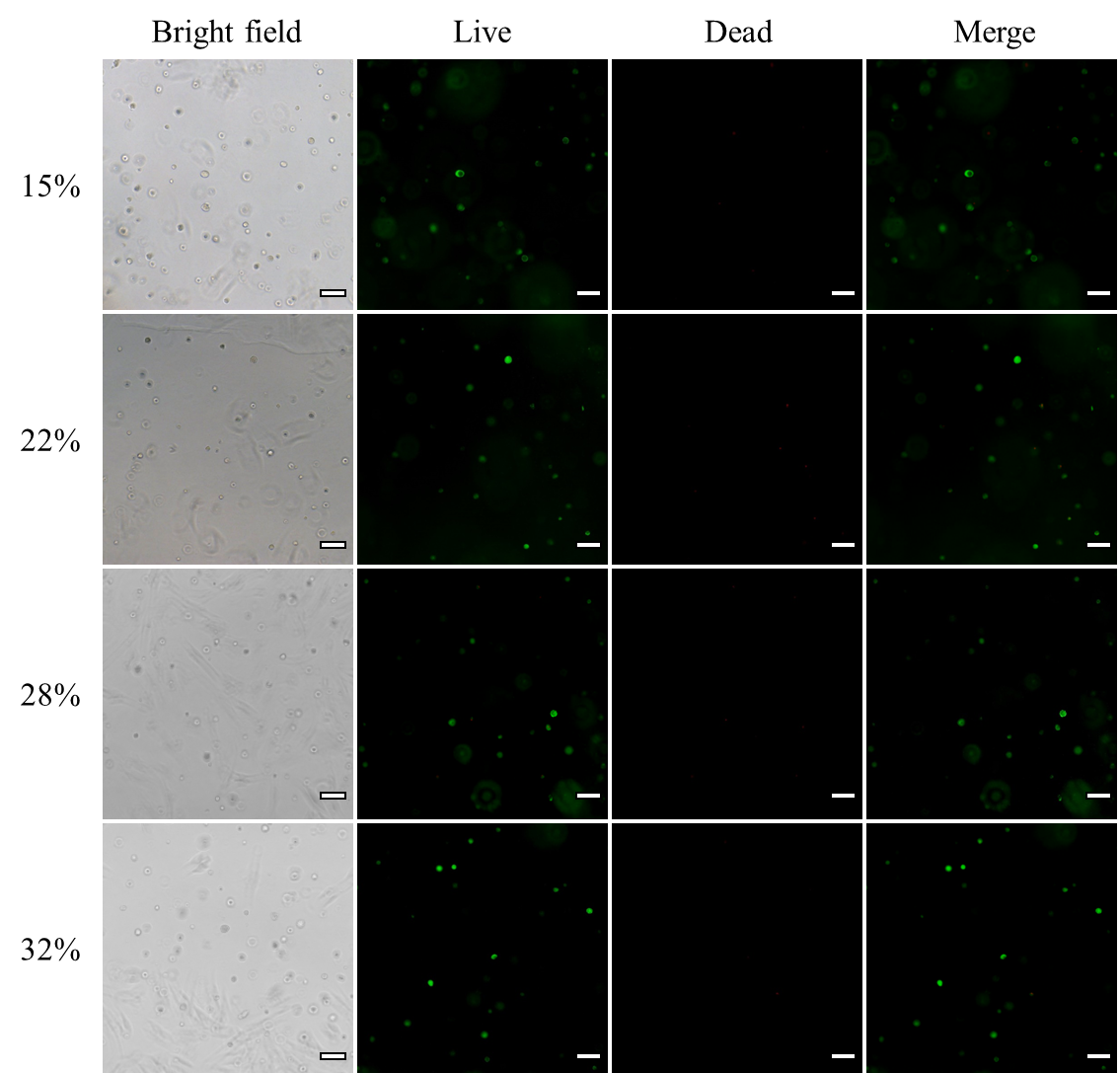


**Fig. S29 Cell biocompatibility of NCGs.** Live/dead staining of hyaline chondrocyte cells after three-dimensional (3D) culture in NCGs for 3 d. The scale bar is 100 μm


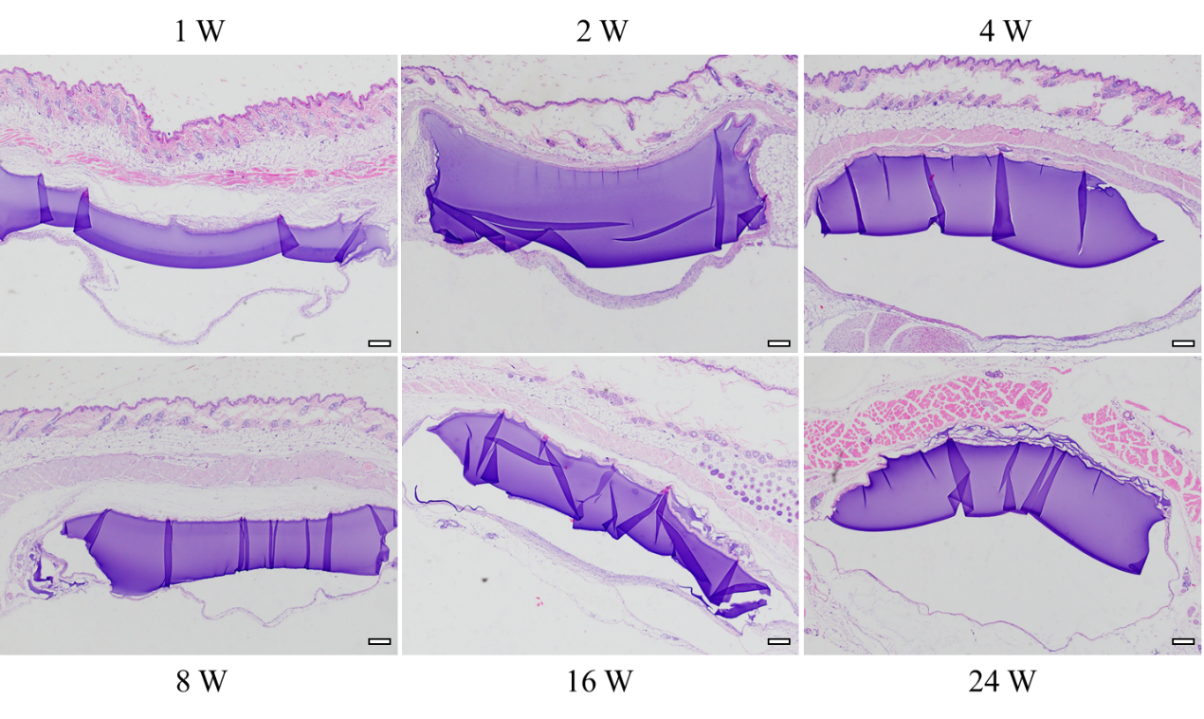


**Fig. S30** **Histological characterization of subcutaneous tissues.** The H&E staining of the subcutaneous tissue with subcutaneous implantation of NCGs at 1, 2, 4, 8, 16, and 24 w. The scale bar is 200 μm


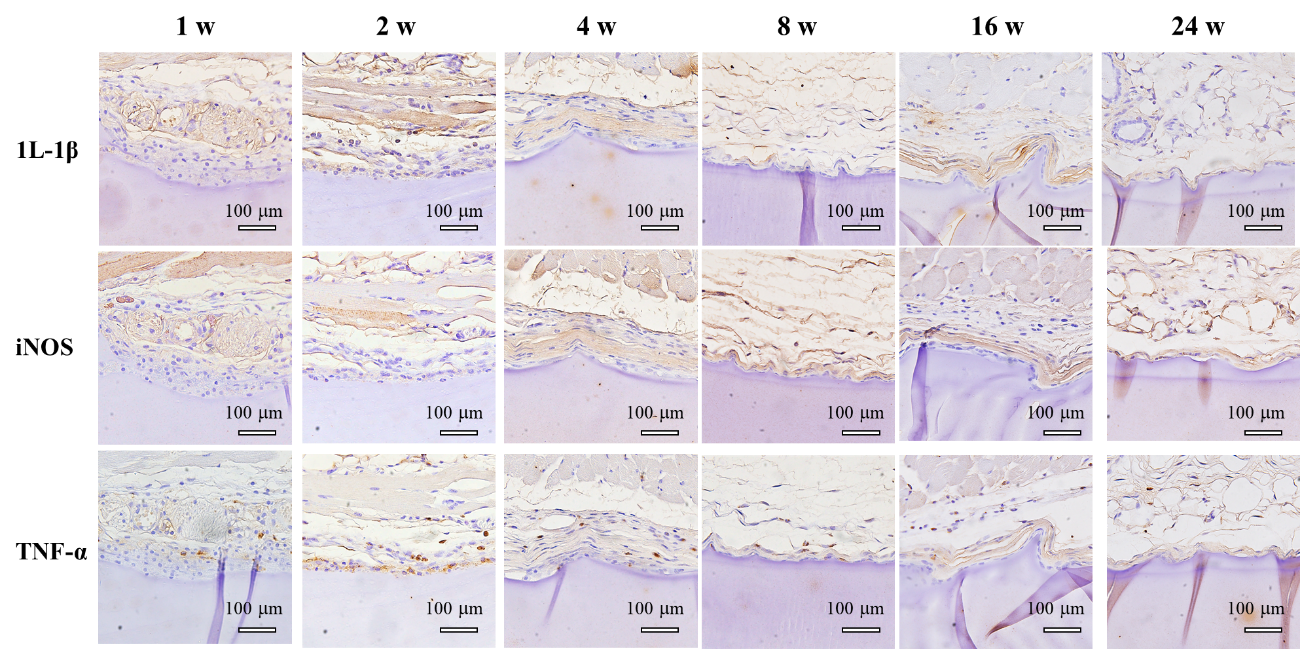


**Fig. S31 Immunohistochemical staining of subcutaneous tissue.** Representative immunohistochemical staining of subcutaneous tissue sections showing the expression of IL-1β, iNOS, and TNF-α at 1, 2, 4, 8, 16, and 24 weeks post-implantation

**Fig. S32 Quantitative Analysis of Inflammatory Response.** Quantification of the positive staining area (%) for IL-1β, iNOS, and TNF-α in subcutaneous tissue at each time point. Error bars represent standard deviations based on three independent measurements


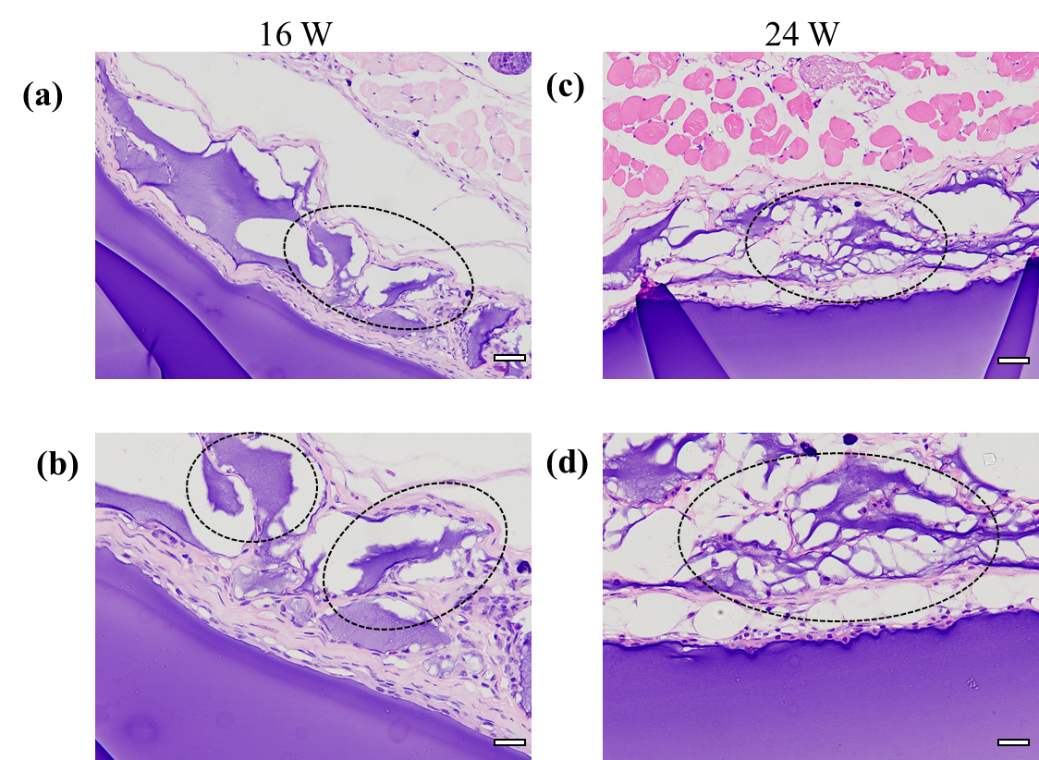


**Fig. S33 Histological characterization of subcutaneous tissues.** The H&E staining of the subcutaneous tissue with subcutaneous implantation of NCGs at 16 (**a, b**) and 24 w (**c, d**). The scale bar in (**a**) and (**c**) is 50 μm. The scale bar in (**b**) and (**d**) is 20 μm


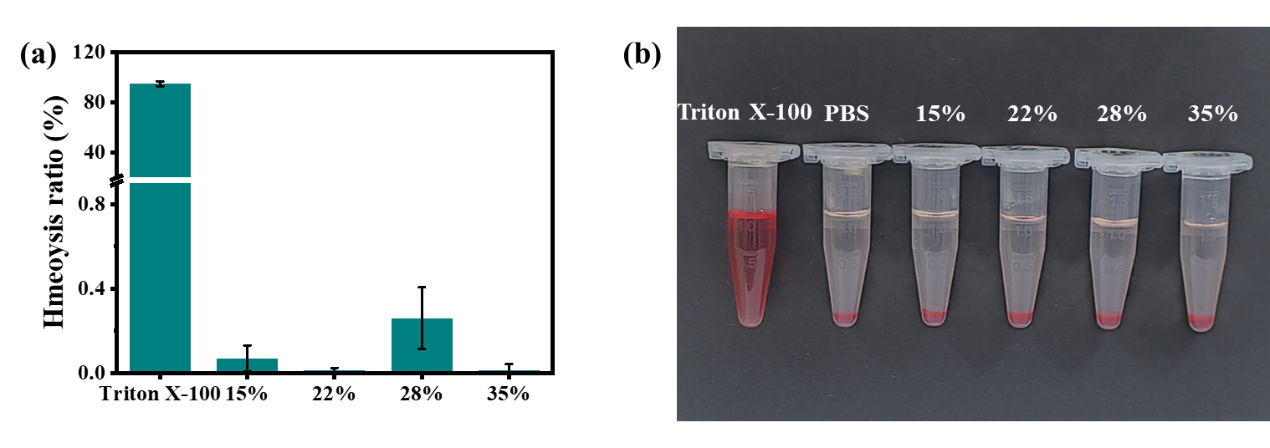


**Fig. S34 Blood compatibility of NCGs.** (**a**) Hemolysis ratio of NCGs and control group. (**b**) Photographs of supernatants of RBCs suspensions treated with Triton X-100, PBS, and NCGs prepared from HAMA NPs with DS of 15%, 22%, 28%, and 35%. Error bars represent standard deviations based on three independent measurements


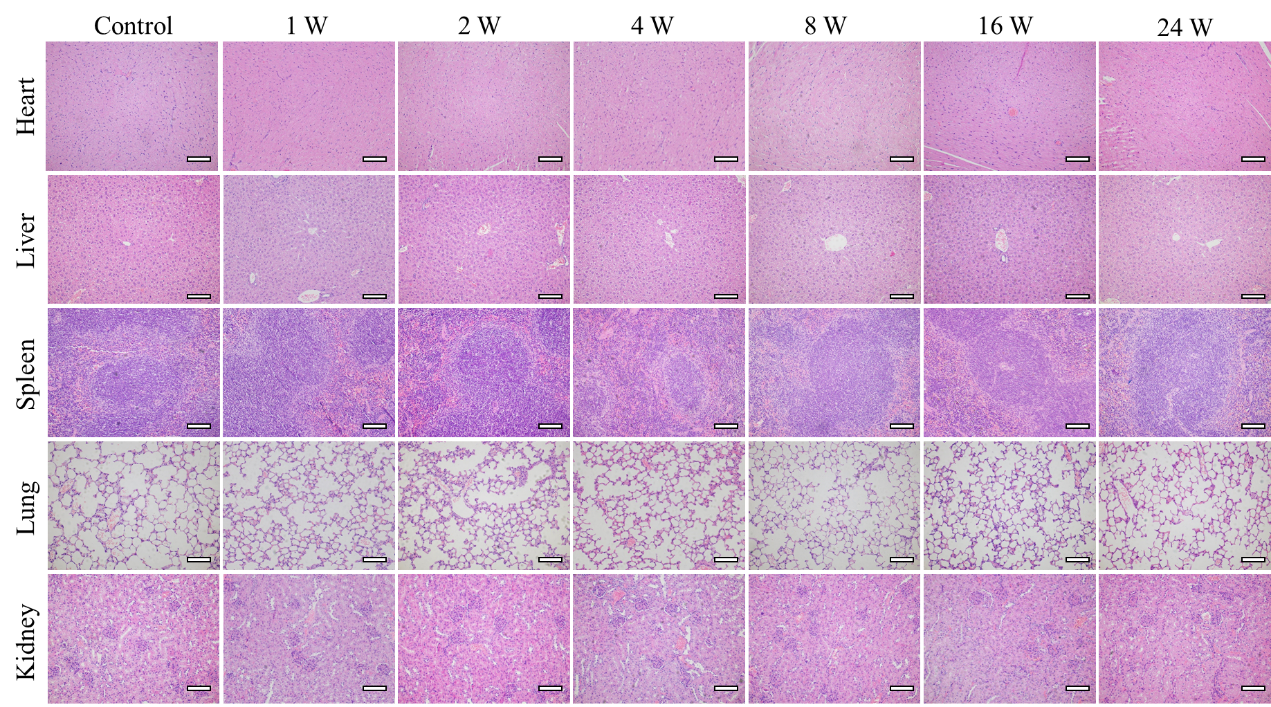


**Fig. S35** **Biocompatibility of NCGs in vivo.** H&E staining of organs after the subcutaneous implantation of NCGs for 1, 2, 4, 8, 16, and 24 w

**Supplementary Tables**

**Table** **S1** The elemental analysis of HAMA NPs with different DS. ADH/-COOH represents the ratio between the added adipic dihydrazide (ADH) and the carboxyl groups (-COOH) of HAMA

|  | **N%** | **C%** | **H%** |
| --- | --- | --- | --- |
| HAMA-15 NPs | 6.41 | 47.19 | 6.51 |
| HAMA-22 NPs | 6.05 | 48.20 | 6.46 |
| HAMA-28 NPs  HAMA-35 NPs | 5.99  5.95 | 47.68  48.24 | 6.39  5.94 |
| ADH/-COOH = 10% | 3.37 | 40.60 | 6.26 |
| ADH/-COOH = 50% | 4.82 | 42.86 | 6.68 |

**Table** **S2** The crosslinking degree in HAMA NPs by ADH with different DS calculated by elemental analysis

|  | **Crosslinking degree of HAMA NPs (%)** |
| --- | --- |
| HAMA-15 NPs | 57.59 |
| HAMA-22 NPs | 55.57 |
| HAMA-28 NPs | 58.76 |
| HAMA-35 NPs | 62.30 |
| ADH/-COOH = 10% | 5.62 |
| ADH/-COOH = 50% | 33.77 |

**Table** **S3** Radius of gyration (R_g_) of the nanophase separation and radius of spherical segregated regions determined by fitting the SAXS data. HAMA NPs-UV represents the individual HAMA NPs after photocrosslinking

|  | **Rg (nm)** | **R (nm)** |
| --- | --- | --- |
| HAMA-15 NPs | 0.67 ± 0.01 | 0.86 ± 0.013 |
| HAMA-22 NPs | 0.68 ± 0.01 | 0.88 ± 0.0113 |
| HAMA-28 NPs | 0.65 ± 0.01 | 0.84 ± 0.0113 |
| HAMA-35 NPs | 0.71 ± 0.01 | 0.92 ± 0.0113 |
| HAMA-15 NPs-UV | 0.53 ± 0.01 | 0.68 ± 0.013 |
| HAMA-22 NPs-UV | 0.62 ± 0.01 | 0.80 ± 0.013 |
| HAMA-28 NPs-UV | 0.66 ± 0.01 | 0.85 ± 0.013 |
| HAMA-35 NPs-UV | 0.75 ± 0.04 | 0.97 ± 0.052 |
| NCG-15 | 0.81 ± 0.02 | 1.05 ± 0.013 |
| NCG-22 | 0.85 ± 0.01 | 1.09 ± 0.0113 |
| NCG-28 | 0.82 ± 0.01 | 1.06 ± 0.0113 |
| NCG-35 | 0.85 ± 0.01 | 1.09 ± 0.0113 |

**Table** **S4** Crosslinking density and water content in NCGs made from 25 mg mL^-1^ HAMA NPs with different DS

|  | **NCG-15** | **NCG-22** | **NCG-28** | **NCG-35** |
| --- | --- | --- | --- | --- |
| Water content (%) | 97.7 | 97.5 | 97.8 | 98.3 |
| Crosslinking density (mol m^-3^) | 53.0 ± 1.6 | 131.2 ± 15.8 | 184.2 ± 13.6 | 192.1 ± 6.3 |

**Table S5** A comparative analysis of the swelling behavior of NCGs and representative non-swelling hydrogels reported in the literature

| **Hydrogel type** | **Swelling ratio** | **Mechanism and principle** | **References** |
| --- | --- | --- | --- |
| NCGs | nonswelling | hydrophobic nanophase domains | This study |
| PAACP hydrogel | ~ 120% | hydrophobic polymer chains | [S10] |
| PVA-CY-ANF hydrogel | ~ 100% | hydrophobic fillers | [S11] |
| Mxene hydrogel | ~ 104% | PVA crystalline microdomains and hydrophobic fillers | [S12] |
| RAAS hydrogel | ~ 101% | thermoresponsive polymers | [S13] |
| Brush polymer hydrogel | ~ 104% | brush architecture with extended chain conformations | [S14] |
| Zwitterionic hydrogel | ~ 118% | hydrophobic interaction and high crosslinking | [S15] |
| PVA exogels | ~ 102% | strong hydrogel crosslinking | [S16] |
| IEDDA HA gels | ~ 101% | strong hydrogel crosslinking | [S17] |
| Supramolecular hydrogel | ~ 100% | hydrophobic interaction and high crosslinking | [S18] |
| PAAc/Gelatin hydrogel | ~ 108% | dense hydrogen bonds | [S19] |

**Table S6** A comparative analysis of the lubrication properties of NCGs and representative lubricative hydrogels reported in the literature

| **Hydrogel type** | **COF** | **References** |
| --- | --- | --- |
| NCGs | ~ 0.002 | This study |
| MS-SLH | ~ 0.0079 | [S20] |
| PVA/PEG-BPP hydrogel | 0.05-0.1 | [S21] |
| DAGQD@Cu@KGN-SAN hydrogel | ~ 0.157 | [S22] |
| Polyelectrolyte hydrogel | ~ 0.16 | [S23] |
| PAA-Fe/PAM hydrogel | ~ 0.0036 | [S24] |
| GelMA-HMS hydrogel | ~ 0.017 | [S25] |
| Electro-responsive supramolecular hydrogel | ~ 0.062 | [S26] |
| SB-modified hyaluronic acid hydrogel | ~ 0.034 | [S27] |

**Table** **S7** Histological grading scale for inflammatory immune response

| (1) Capsule quality | Score |
| --- | --- |
| Capsule is fibrous, mature, not dense, resembling connective or fat tissue in the non-injured regions | 5 |
| Capsule tissue is fibrous but immature, showing fibroblasts and little collagen | 4 |
| Capsule tissue is granulous or dense, containing mainly fibroblasts and some inflammatory cells | 3 |
| Capsule tissue is granulous or dense, containing both fibroblasts and many inflammatory cells | 2 |
| Capsule consists of masses of inflammatory cells with little or no signs of connective tissue organization | 1 |
| Cannot be evaluated because of infection or other factors not necessarily related to the material | 0 |
| (2) Capsule thickness | Score |
| 1−4 fibroblasts | 5 |
| 5−9 fibroblasts | 4 |
| 10−30 fibroblasts with few inflammatory cells | 3 |
| 10−30 containing both fibroblasts and inflammatory cells | 2 |
| 10−30 inflammatory cells with few fibroblasts | 1 |
| not applicable | 0 |
| (3) Cell infiltration | Score |
| Fibroblasts contact the implant surface without the presence  of macrophages or leucocytes | 4 |
| Scattered foci of macrophages and leucocytes are present | 3 |
| One layer of macrophages and leucocytes are present | 2 |
| Multiple layers of macrophages and leucocytes present | 1 |
| Cannot be evaluated because of infection or other factors  not necessarily related to the material | 0 |

**Table** **S8** Histological grading of NCGs after 1, 2, 4, 8, 16, and 24 w of subcutaneous implantation

| Implantation time | Capsule quality | Capsule thickness | Cell infiltration |
| --- | --- | --- | --- |
| 1 w | 2.83 ± 0.41 | 3.67 ± 0.52 | 2.67 ± 0.52 |
| 2 w | 2.83 ± 0.41 | 3.67 ± 0.52 | 2.83 ± 0.41 |
| 4 w | 3.33 ± 0.52 | 4.00 | 3.17 ± 0.41 |
| 8 w | 3.50 ± 0.55 | 4.17 ± 0.41 | 3.00 |
| 16 w  24 w | 3.67 ± 0.52  3.67 ± 0.52 | 4.00  4.33 ± 0.52 | 3.50 ± 0.55  3.50 ± 0.55 |

**Supplementary References**

1. Y. Diao, Y. Zhou, T. Kurosawa, L. Shaw, C. Wang et al., Flow-enhanced solution printing of all-polymer solar cells. Nat. Commun. **6**, 7955 (2015). <https://doi.org/10.1038/ncomms8955>
2. W. Wu, H. Feng, L. Xie, A. Zhang, F. Liu et al., Reprocessable and ultratough epoxy thermosetting plastic. Nat. Sustain. **7**(6), 804–811 (2024). <https://doi.org/10.1038/s41893-024-01331-9>
3. J. Pietrasik, B.S. Sumerlin, H.-I. Lee, R.R. Gil, K. Matyjaszewski, Structural mobility of molecular bottle-brushes investigated by NMR relaxation dynamics. Polymer **48**(2), 496–501 (2007). <https://doi.org/10.1016/j.polymer.2006.11.057>
4. M. Wu, A.M. Vartanian, G. Chong, A.K. Pandiakumar, R.J. Hamers et al., Solution NMR analysis of ligand environment in quaternary ammonium-terminated self-assembled monolayers on gold nanoparticles: the effect of surface curvature and ligand structure. J. Am. Chem. Soc. **141**(10), 4316–4327 (2019). <https://doi.org/10.1021/jacs.8b11445>
5. Y.-G. Kim, M. Wagner, H. Thérien-Aubin, Dynamics of soft and hairy polymer nanoparticles in a suspension by NMR relaxation. Macromolecules **53**(3), 844–851 (2020). <https://doi.org/10.1021/acs.macromol.9b01813>
6. T. Kopač, M. Abrami, M. Grassi, A. Ručigaj, M. Krajnc, Polysaccharide-based hydrogels crosslink density equation: a rheological and LF-NMR study of polymer-polymer interactions. Carbohydr. Polym. **277**, 118895 (2022). <https://doi.org/10.1016/j.carbpol.2021.118895>
7. A. Kargerová, M. Pekař, Densitometry and ultrasound velocimetry of hyaluronan solutions in water and in sodium chloride solution. Carbohydr. Polym. **106**, 453–459 (2014). <https://doi.org/10.1016/j.carbpol.2014.01.020>
8. K.Y. Lee, J.A. Rowley, P. Eiselt, E.M. Moy, K.H. Bouhadir et al., Controlling mechanical and swelling properties of alginate hydrogels independently by cross-linker type and cross-linking density. Macromolecules **33**(11), 4291–4294 (2000). <https://doi.org/10.1021/ma9921347>
9. Y. Li, N. Khuu, E. Prince, H. Tao, N. Zhang et al., Matrix stiffness-regulated growth of breast tumor spheroids and their response to chemotherapy. Biomacromolecules **22**(2), 419–429 (2021). <https://doi.org/10.1021/acs.biomac.0c01287>
10. G. Tian, D. Yang, C. Liang, Y. Liu, J. Chen et al., A nonswelling hydrogel with regenerable high wet tissue adhesion for bioelectronics. Adv. Mater. **35**(18), 2212302 (2023). <https://doi.org/10.1002/adma.202212302>
11. H.J. Kim, H. Kim, Y.H. Choi, E.S. Lee, Y.H. Kim et al., Rapid fabrication of tendon-inspired ultrastrong, water-rich hydrogel fibers: synergistic engineering of cyano-*p*-aramid nanofibers and poly(vinyl alcohol). ACS Nano **19**(8), 8316–8327 (2025). <https://doi.org/10.1021/acsnano.4c18686>
12. Y. Zhang, Z. Xu, Y. Yuan, C. Liu, M. Zhang et al., Flexible antiswelling photothermal-therapy MXene hydrogel-based epidermal sensor for intelligent human–machine interfacing. Adv. Funct. Mater. **33**(21), 2300299 (2023). <https://doi.org/10.1002/adfm.202300299>
13. S. Bian, L. Hao, X. Qiu, J. Wu, H. Chang et al., An injectable rapid-adhesion and anti-swelling adhesive hydrogel for hemostasis and wound sealing. Adv. Funct. Mater. **32**(46), 2207741 (2022). <https://doi.org/10.1002/adfm.202207741>
14. F. Jia, J.M. Kubiak, M. Onoda, Y. Wang, R.J. MacFarlane, Design and synthesis of quick setting nonswelling hydrogels *via* brush polymers. Adv. Sci. **8**(16), 2100968 (2021). <https://doi.org/10.1002/advs.202100968>
15. Q. Yu, H. Sun, L. Zhang, L. Jiang, L. Liang et al., A zwitterionic hydrogel with anti-oxidative and anti-inflammatory properties for the prevention of peritoneal adhesion by inhibiting mesothelial-mesenchymal transition. Adv. Healthc. Mater. **12**(30), 2301696 (2023). <https://doi.org/10.1002/adhm.202301696>
16. L. Xu, S. Gao, Q. Guo, C. Wang, Y. Qiao et al., A solvent-exchange strategy to regulate noncovalent interactions for strong and antiswelling hydrogels. Adv. Mater. **32**(52), 2004579 (2020). <https://doi.org/10.1002/adma.202004579>
17. V. Delplace, P.E.B. Nickerson, A. Ortin-Martinez, A.E.G. Baker, V.A. Wallace et al., Nonswelling, ultralow content inverse electron-demand Diels–alder hyaluronan hydrogels with tunable gelation time: synthesis and *in vitro* evaluation. Adv. Funct. Mater. **30**(14), 1903978 (2020). <https://doi.org/10.1002/adfm.201903978>
18. Z. Qin, X. Yu, H. Wu, J. Li, H. Lv et al., Nonswellable and tough supramolecular hydrogel based on strong micelle cross-linkings. Biomacromolecules **20**(9), 3399–3407 (2019). <https://doi.org/10.1021/acs.biomac.9b00666>
19. M. Pi, S. Qin, S. Wen, Z. Wang, X. Wang et al., Rapid gelation of tough and anti-swelling hydrogels under mild conditions for underwater communication. Adv. Funct. Mater. **33**(1), 2210188 (2023). <https://doi.org/10.1002/adfm.202210188>
20. S. Ma, L. Liu, W. Zhao, R. Li, X. Zhao et al., Earthworm inspired lubricant self-pumping hydrogel with sustained lubricity at high loading. Nat. Commun. **16**(1), 398 (2025). <https://doi.org/10.1038/s41467-024-55715-8>
21. J. Liu, W. Zhao, Z. Ma, H. Zhao, L. Ren, Cartilage-bioinspired tenacious concrete-like hydrogel verified *via* *in situ* testing. Nat. Commun. **16**(1), 2309 (2025). <https://doi.org/10.1038/s41467-025-57653-5>
22. H. He, Q. Zhang, Y. Zhang, S. Qu, B. Li et al., Injectable bioadhesive and lubricating hydrogel with polyphenol mediated single atom nanozyme for rheumatoid arthritis therapy. Nat. Commun. **16**(1), 2768 (2025). <https://doi.org/10.1038/s41467-025-58059-z>
23. Z. Wang, P. Li, B. Li, S. Yang, T. Zhao et al., Innovating lubrication with polyelectrolyte hydrogels: sustained performance through lipid dynamics. Adv. Funct. Mater. **35**(3), 2413712 (2025). <https://doi.org/10.1002/adfm.202413712>
24. Z. Zhang, Y. Ma, B. Yu, S. Ma, H. Yang et al., Vertical and horizontal double gradient design for super-slippery and high-bearing hydrogel skeleton. Adv. Funct. Mater. **34**(29), 2400360 (2024). <https://doi.org/10.1002/adfm.202400360>
25. X. He, S. He, G. Xiang, L. Deng, H. Zhang et al., Precise lubrication and protection of cartilage damage by targeting hydrogel microsphere. Adv. Mater. **36**(40), 2405943 (2024). <https://doi.org/10.1002/adma.202405943>
26. J. Kang, X. Zhang, X. Yang, X. Yang, S. Wang et al., Mucosa-inspired electro-responsive lubricating supramolecular-covalent hydrogel. Adv. Mater. **35**(46), e2307705 (2023). <https://doi.org/10.1002/adma.202307705>
27. J. Hou, Y. Lin, C. Zhu, Y. Chen, R. Lin et al., Zwitterion-lubricated hydrogel microspheres encapsulated with metformin ameliorate age-associated osteoarthritis. Adv. Sci. **11**(30), e2402477 (2024). <https://doi.org/10.1002/advs.202402477>
